# Haptoglobin Phenotypes Stratify Post-Exertional Cognitive Dysfunction Associated with Altered Cerebral Oxygenation and Metabolic Signatures in Long COVID

**DOI:** 10.3390/ijms27157000

**Published:** 2026-08-04

**Authors:** Atefeh Moezzi, Wesam Elremaly, Corinne Leveau, Anita Franco, Oleg Nepotchatykh, Christopher W. Armstrong, Alain Moreau

**Affiliations:** 1 Department of Biochemistry and Molecular Medicine, Faculty of Medicine, Université de Montréal, Montreal, QC H3C 3J7, Canada; 2 Viscogliosi Laboratory in Molecular Genetics of Musculoskeletal Diseases, Azrieli Research Center, CHU Sainte-Justine, Montreal, QC H3T 1C5, Canada; 3 Open Medicine Foundation ME/CFS Collaborative Center, CHU Sainte-Justine/Université de Montréal, Montreal, QC H3T 1C5, Canada; 4 ICanCME Research Network, Azrieli Research Center, CHU Sainte-Justine, Montreal, QC H3T 1C5, Canada; 5 PO-Laboratories, Montreal, QC H4T 2A3, Canada; 6 Department of Biochemistry and Molecular Biology, Bio21 Molecular Science and Biochemistry Institute, University of Melbourne, Parkville, VIC 3010, Australia; 7 Department of Stomatology, Faculty of Dentistry, Université de Montréal, Montreal, QC H3C 3J7, Canada

**Keywords:** long COVID, post-COVID-19 condition, haptoglobin phenotypes, post-exertional malaise, cognition, cerebral oxygenation, metabolomics, biomarkers, precision medicine

## Abstract

Long COVID (LC) is a heterogeneous post-infectious syndrome characterized by persistent symptoms, yet the biological basis underlying its interindividual variability remains poorly understood. Given the clinical overlap between LC and myalgic encephalomyelitis (ME), and prior demonstration that haptoglobin (Hp) phenotypes modulate symptom severity in ME, we investigated whether Hp phenotypes similarly stratify post-exertional cognitive dysfunction in LC. In this longitudinal observational study, 44 individuals with LC and 20 short-course COVID controls, who recovered rapidly from SARS-CoV-2 infection without persistent symptoms or sequelae, underwent Hp phenotyping alongside metabolomic and physiological profiling before and after a standardized 90 min passive post-exertional challenge. Hp phenotypes identified clinically distinct LC subgroups. Compared with Hp1-1 individuals, Hp2 allele carriers exhibited greater fatigue, poorer physical function, and more severe post-exertional symptoms. Immediately following the challenge, Hp2-2 participants with LC showed significant cognitive decline, whereas Hp1-1 individuals demonstrated cognitive resilience and more favorable longitudinal cognitive trajectories. This differential susceptibility was accompanied by higher post-exertional cerebral fractional tissue oxygen extraction in the right hemisphere in Hp1-1 individuals and by distinct metabolic signatures, with Hp2 allele carriers exhibiting lower post-exertional plasma concentrations of citric acid, isethionate, and glucosamine. Lower metabolite levels were associated with poorer cognitive performance. These findings support Hp phenotypes as promising candidate biomarkers for biological stratification in Long COVID, pending validation in larger independent cohorts.

## 1. Introduction

Long COVID (LC), also referred to as post-COVID-19 condition, post-acute sequelae of SARS-CoV-2 infection (PASC), or long-haul COVID, has emerged as a major global health challenge. It is defined by the World Health Organization as symptoms that arise or persist at least three months after SARS-CoV-2 infection and last for a minimum of two months in the absence of an alternative diagnosis [1]. LC affects an estimated 30–40% of infected individuals worldwide [2]. Although fatigue, cognitive dysfunction, autonomic disturbances, sleep abnormalities, and post-exertional malaise (PEM) are among its most disabling manifestations [3,4], disease severity and recovery trajectories vary markedly between patients, highlighting the need to identify biological factors that underlie this heterogeneity.

The close clinical resemblance between LC and myalgic encephalomyelitis (ME), particularly the presence of PEM and cognitive dysfunction, suggests that both conditions may share common pathophysiological mechanisms. Increasing evidence implicates oxidative stress, mitochondrial dysfunction, endothelial abnormalities, and metabolic dysregulation in both post-infectious syndromes, yet these mechanisms alone do not explain why some individuals develop severe post-exertional deterioration whereas others remain relatively resilient. Identifying host-specific modifiers that influence vulnerability to physiological stress is therefore essential for improving biological stratification and advancing precision medicine approaches.

Haptoglobin (Hp) is a multifunctional plasma glycoprotein involved in hemoglobin (Hb) scavenging, oxidative stress regulation, and vascular homeostasis. Its three common phenotypes (Hp1-1, Hp2-1, and Hp2-2) differ substantially in antioxidant capacity, inflammatory regulation, and vascular protection, with the Hp2 phenotypes generally exhibiting reduced functional efficiency. In our previous work, we demonstrated that Hp phenotypes modulate PEM severity and cognitive dysfunction in ME, identifying Hp2 allele carriers as being more susceptible to post-exertional deterioration and revealing post-exertional alterations in circulating Hp and its molecular organization [5]. These findings raise the possibility that genetically determined differences in Hp function may similarly contribute to the biological heterogeneity observed in LC, while the functional expression of this inherited background may differ between relatively early and long-standing post-infectious disease states.

Building on these observations, we investigated whether Hp phenotypes stratify susceptibility to post-exertional cognitive dysfunction in LC and whether this vulnerability is accompanied by distinct cerebral oxygenation and metabolic signatures. To address this question, we integrated clinical symptom profiling, longitudinal cognitive assessment, cerebral oxygenation measurements using near-infrared spectroscopy (NIRS), and plasma biomarkers as well as metabolomic profiling before and after a standardized passive post-exertional challenge. By identifying biologically distinct patient subgroups, this study aims to evaluate the translational potential of Hp phenotyping as a candidate biomarker for biological stratification in LC, with potential implications for future precision medicine approaches.

## 2. Results

### 2.1. Study Population and Hp Phenotype Distribution

A total of 64 participants were included in this study, comprising 44 individuals with LC and 20 individuals who recovered from SARS-CoV-2 infection within two weeks, referred to as short-course COVID (SC). The baseline demographic and clinical characteristics of both cohorts are summarized in Table 1.

The LC cohort was predominantly female (42 females and 2 males), as was the SC cohort (14 females and 6 males). Mean age and BMI did not differ significantly between the two groups. In contrast, participants with LC exhibited a substantially greater symptom burden than SC controls across all validated clinical questionnaires. 36-Item Short Form Health Survey (SF-36) physical and mental health scores were markedly reduced in the LC group (both *p* < 0.0001), while Multidimensional Fatigue Inventory-20 (MFI-20) scores demonstrated significantly higher levels of general, physical, and mental fatigue, reduced activity, and reduced motivation. Similarly, DePaul Symptom Questionnaire (DSQ) scores revealed significantly greater neuroendocrine/autonomic/immune dysfunction, cognitive dysfunction, PEM, and sleep disturbances in LC participants compared with SC controls (all *p* < 0.0001) (Table 1).

Hp phenotype distribution was comparable between LC and SC participants (Table 2). Overall, these findings indicate that although LC and SC participants exhibited similar demographic characteristics and comparable Hp phenotype distributions, individuals with LC experienced substantially greater clinical impairment. The balanced distribution of Hp phenotypes across groups provided an appropriate control framework for investigating disease-specific mechanisms linked to inherited Hp variants. This distribution also minimized the likelihood that differences in phenotype frequency accounted for the observed heterogeneity of post-exertional responses observed in LC. Within the LC cohort, baseline characteristics were further examined according to Hp phenotype; BMI was comparable across all subgroups. However, a modest difference in baseline age was observed. Specifically, Hp2-1 participants were younger than Hp1-1 individuals, while no significant age difference was detected between Hp1-1 and Hp2-2 groups. Documenting these minor intra-cohort variations provides the necessary baseline control data for the adjusted multiple linear regression modeling used throughout the study.

### 2.2. Association Between Hp Phenotypes and Symptom Severity

**Figure 1:**
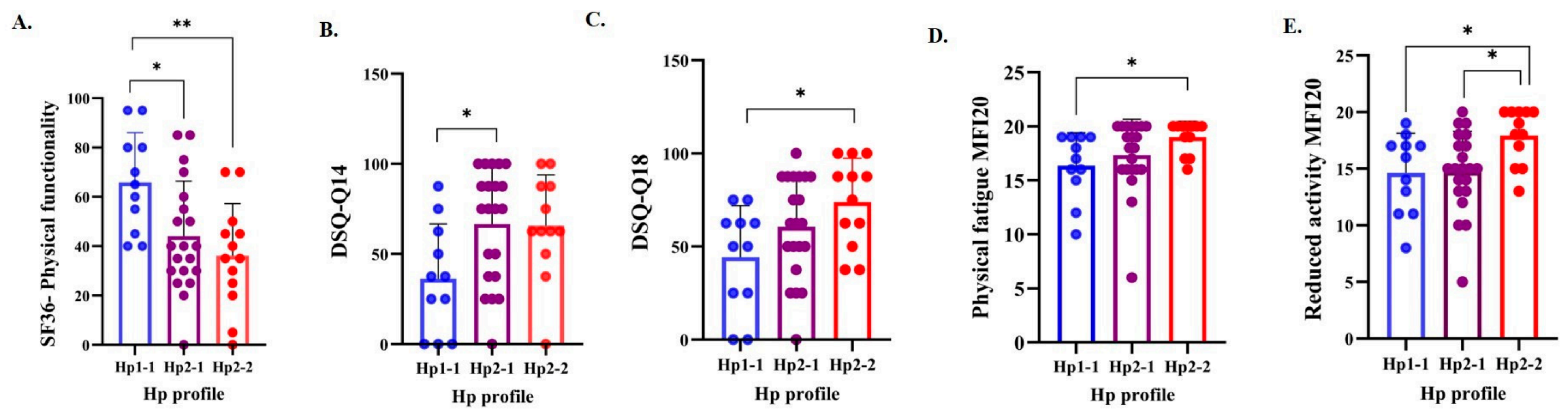
Symptom severity across Hp phenotypes in patients with LC. (**A**) Symptom severity based on the SF-36 physical functionality score. Patients with either Hp2-1 (purple) or Hp2-2 (red) phenotypes exhibited more severe symptoms than those with Hp1-1 phenotype (blue). (**B**,**C**) Symptom severity based on DSQ Q14 and Q18, which evaluate “dead, heavy feeling after starting to exercise” and “physically drained or sick after mild activity,” respectively. Individuals with the Hp1-1 phenotype reported lower symptom severity compared with Hp2 carriers, with Hp2-1 associated with the highest severity for Q14 and Hp2-2 for Q18. (**D**,**E**) Symptom severity based on MFI20 physical fatigue and reduced activity scores indicates more severity in Hp2 carriers. All data are represented as mean ± standard error of the mean. One-way ANOVA was performed to assess significant differences among the normally distributed groups, followed by the Tukey post hoc test for multiple comparisons. Kruskal–Wallis test followed by Dunn’s test for multiple comparisons was used for non-normally distributed groups. Results were considered significant at * *p* value < 0.05 and ** *p*-value  <  0.01.

Given the marked clinical heterogeneity observed among individuals with LC, we next investigated whether symptom severity differed according to Hp phenotypes. Analysis of SF-36 physical function scores revealed significant differences across Hp phenotype groups (one-way ANOVA, *p* = 0.02; η^2^ = 0.23), with Hp2 carriers exhibiting poorer physical functioning than individuals with the Hp1-1 phenotype (Figure 1A).

Assessment of PEM using the DSQ further demonstrated variation in symptom severity across Hp phenotypes. Both DSQ items Q14 (“dead, heavy feeling after starting to exercise”) and Q18 (“physically drained or sick after mild activity”) differed significantly among Hp phenotypes (*p* = 0.02 for both comparisons), with large effect sizes (η^2^ = 0.16 and η^2^ = 0.15, respectively). Hp1-1 individuals consistently reported lower symptom severity, whereas Hp2 carriers experienced greater exertional intolerance. Notably, the Hp2-1 phenotype was associated with the highest Q14 scores, while Hp2-2 individuals exhibited the greatest severity for Q18 (Figure 1B,C).

A similar pattern was observed for fatigue-related outcomes. MFI-20 analyses identified significant differences in physical fatigue (*p* = 0.02; η^2^ = 0.13) and reduced activity (*p* = 0.04; η^2^ = 0.16), with participants carrying the Hp2-2 phenotype reporting the greatest impairment (Figure 1D,E). Collectively, these findings indicate that the phenotypes with Hp2 are associated with a more severe clinical presentation characterized by reduced physical function, increased fatigue, and heightened susceptibility to PEM.

To determine whether these associations were independent of potential confounding factors, multiple linear regression analyses were performed, adjusting for age, sex, BMI and disease duration (Supplementary Table S1). The resulting models explained a significant proportion of the variance in SF-36 physical function (R^2^ = 0.30, *p* = 0.015), MFI-20 physical fatigue (R^2^ = 0.28, *p* = 0.0213), and DSQ PEM scores (R^2^ = 0.28, *p* = 0.025). Across all three models, Hp phenotypes emerged as a significant independent predictor. Progression from Hp1-1 toward Hp2-2 was associated with reduced physical functioning (β = −14.8, *p* = 0.004) and with increased physical fatigue (β = 3.4, *p* = 0.008) and PEM severity (β = 29.5, *p* = 0.013). In contrast, age, sex, BMI, and disease duration did not significantly contribute to any of the regression models (*p* > 0.05), indicating that the observed associations were not explained by these clinical or demographic variables. Together, these findings demonstrate that Hp phenotype is independently associated with physical function, fatigue, and post-exertional symptom severity in LC, supporting its potential value as a candidate marker of disease heterogeneity.

### 2.3. Cognitive Responses to Post-Exertional Stress

Given the independent association between Hp phenotypes and symptom severity, we next investigated whether Hp phenotypes also influenced susceptibility to post-exertional cognitive dysfunction. Cognitive performance was assessed using the BrainCheck platform at baseline (T0) and immediately after the passive post-exertional challenge (T90), as well as longitudinally at baseline over the three sequential study visits.

#### 2.3.1. Acute Post-Exertional Cognitive Responses

At baseline, no significant differences in global cognitive performance were observed either between LC and SC participants or among Hp phenotype groups within the LC cohort. However, following the post-exertional challenge, distinct cognitive trajectories emerged. Whereas SC participants demonstrated the expected improvement in performance consistent with a practice effect, LC participants showed an opposite pattern characterized by post-exertional cognitive decline, suggesting impaired cognitive adaptability in response to physiological stress (Supplementary Table S2). Although the delayed clinical manifestations of PEM typically peak 24–48 h after exertion, these findings indicate that measurable alterations in cognitive performance can already be detected immediately following the prolonged passive post-exertional challenge.

**Figure 2:**
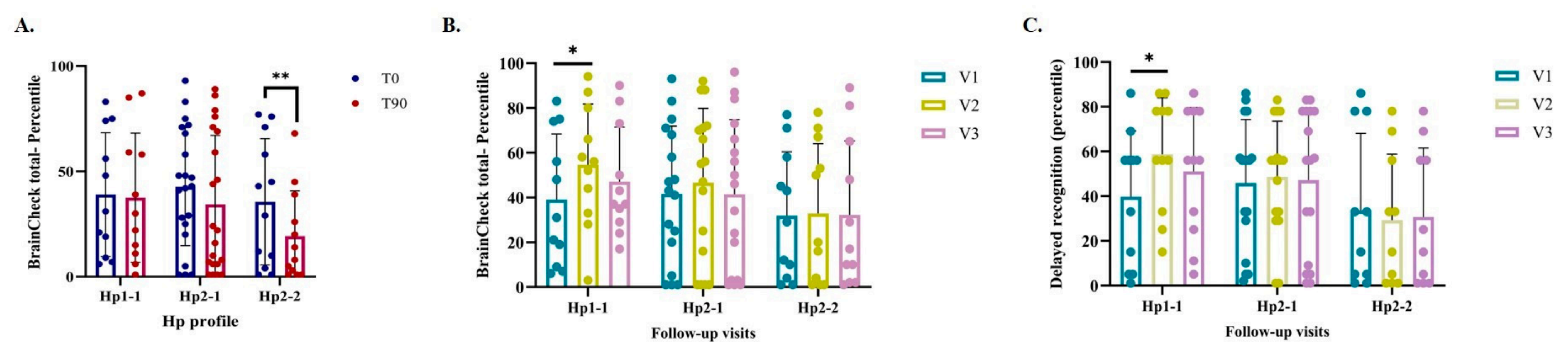
Cognitive assessment in LC individuals with different Hp phenotypes. (**A**) BrainCheck analysis demonstrated a significant reduction in total BrainCheck scores following the post-exertional challenge in patients with the Hp2-2 phenotype. In contrast, no significant baseline differences were observed among the three phenotype groups, and no significant post-PEM cognitive decline was detected in the Hp1-1 and Hp2-1 phenotypes. (**B**,**C**) BrainCheck analysis across the three follow-up visits indicated a significant increase in “total BrainCheck scores” as well as “delayed recognition” in patients with the Hp1-1 phenotype, whereas cognitive performance remained unchanged in Hp2 carriers. Paired *t*-tests were performed to assess within-group changes at T0 vs. T90. Data distribution was assessed prior to analysis. For comparisons among three independent groups, one-way ANOVA was used for normally distributed variables, followed by Tukey’s multiple comparisons test. For non-normally distributed data, the Kruskal–Wallis test was applied, followed by Dunn’s multiple comparisons test. For repeated-measures analyses, normally distributed data were analyzed using repeated-measures ANOVA (RM-ANOVA), followed by Tukey’s multiple comparisons test when appropriate. Non-normally distributed repeated-measures data were analyzed using the Friedman test, followed by Dunn’s multiple comparisons test. Results were considered significant at * *p*-value < 0.05 and ** *p*-value < 0.01.

Stratification by Hp phenotype further revealed distinct post-exertional cognitive responses across Hp groups. Individuals with the Hp2-2 phenotype exhibited a significant reduction in total BrainCheck scores at T90 compared with baseline (paired *t*-test, *p* < 0.05; Cohen’s *d* = 0.9). This large effect size underscores the clinical significance of this cognitive decline within the Hp2-2 subgroup (Figure 2A). In contrast, no significant post-exertional cognitive decline was observed among participants with Hp1-1 or Hp2-1 phenotypes, indicating that the immediate cognitive consequences of physiological stress are not uniformly distributed across Hp subgroups.

#### 2.3.2. Domain-Specific Cognitive Analyses

To determine whether the observed differences in overall BrainCheck performance across Hp phenotypes were driven by specific cognitive domains, individual BrainCheck subtests were analyzed at baseline and immediately following the passive post-exertional challenge (Supplementary Figure S2). These analyses included attention, executive function (mental flexibility and Stroop), digital symbol recognition, immediate recognition, and delayed recognition. Cross-sectional comparisons of individual cognitive domains among Hp phenotype groups revealed no statistically significant differences at either baseline or T90. However, these analyses assess absolute performance at individual time points and are distinct from the within-participant response to post-exertional stress. Notably, the significant post-exertional decline in the total BrainCheck composite score observed in individuals with the Hp2-2 phenotype indicates greater global cognitive vulnerability to the post-exertional challenge.

#### 2.3.3. Longitudinal Cognitive Trajectories

Longitudinal analyses across the three study visits further supported these distinct cognitive trajectories across Hp phenotypes. Participants carrying the Hp1-1 phenotype demonstrated significant improvements in total BrainCheck scores over time, accompanied by enhanced delayed recognition performance (repeated-measures ANOVA, η^2^ = 0.28 and η^2^ = 0.27, respectively) (Figure 2B,C). No comparable longitudinal improvement was observed among Hp2 carriers, whose cognitive performance remained largely unchanged throughout follow-up.

To further examine long-term cognitive trajectories, LC participants were classified as deteriorated, stable, or improved according to changes in BrainCheck composite scores between the first (V1) and third (V3) visits (Table 3). Hp1-1 individuals were markedly underrepresented among participants with deteriorating cognition, accounting for only 10% (*n* = 1) of this subgroup, compared with approximately one-third of participants exhibiting stable (31.6%, *n* = 6) or improving (30.8%, *n* = 4) cognitive trajectories. Conversely, Hp2 phenotypes, comprising both Hp2-1 and Hp2-2, predominated among individuals experiencing longitudinal cognitive decline, accounting for a combined 90.0% (*n* = 9) of the deteriorated subgroup.

These findings suggest that, whereas acute post-exertional cognitive vulnerability was most evident in the Hp2-2 subgroup, unfavorable longitudinal cognitive trajectories were more broadly associated with Hp2 allele carriage, while Hp1-1 was associated with relative cognitive resilience.

#### 2.3.4. Summary of Cognitive Findings

Taken together, both the acute post-exertional response and longitudinal follow-up consistently identified Hp2-2 as the phenotype most susceptible to cognitive deterioration, whereas Hp1-1 was associated with relative cognitive resilience. These observations position Hp phenotyping as a promising biomarker for biological stratification of post-exertional cognitive vulnerability in LC, pending validation in larger independent cohorts.

### 2.4. Cerebral Oxygen Extraction Differences According to Hp Phenotypes

Given the distinct post-exertional cognitive responses observed across Hp phenotypes, we next investigated whether these differences were accompanied by alterations in cerebral oxygen utilization. Cerebral fractional tissue oxygen extraction (cFTOE) was assessed using NIRS in a subset of participants (LC, *n* = 22; SC, *n* = 19).

**Figure 3:**
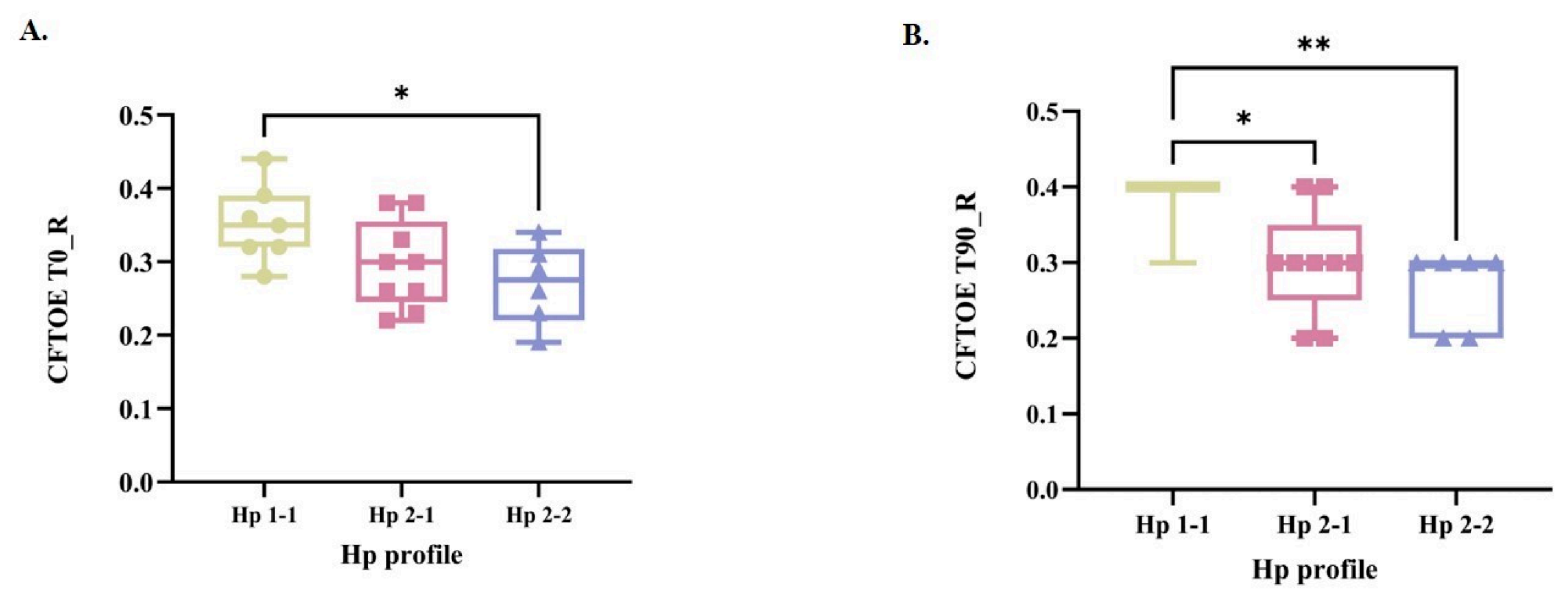
Cerebral fractional tissue oxygen extraction (cFTOE) across Hp phenotypes in LC patients. (**A**,**B**) Significant differences in cFTOE across Hp phenotypes were observed in the right hemisphere. At baseline, cFTOE was lower in the Hp2-2 group compared with Hp1-1, while at T90, cFTOE was reduced in both Hp2-1 and Hp2-2 groups relative to Hp1-1, indicating greater post-exertional alterations in Hp2 carriers. One-way ANOVA was performed to assess significant differences among the three groups, followed by the Tukey post hoc test for multiple comparisons. Graphs for phenotypes Hp1-1, Hp2-1 and Hp2-2 are represented by the colors pale green, mauve and blue respectively. Results were considered significant at * *p*-value < 0.05 and ** *p*-value  <  0.01.

Within the LC cohort, statistically significant differences in cFTOE across Hp phenotypes were detected in the right cerebral hemisphere (A2). At baseline under resting conditions, individuals with the Hp1-1 phenotype exhibited significantly higher cFTOE values than those with the Hp2-2 phenotype (*p* < 0.05; Cohen’s *d* = 0.82), indicating greater cFTOE under resting conditions (Figure 3A). Following the post-exertional challenge, these differences became more pronounced, with Hp1-1 participants maintaining significantly higher cFTOE values than both Hp2-1 (Cohen’s *d* = 1.16) and Hp2-2 (Cohen’s *d* = 1.25) groups (one-way ANOVA, η^2^ = 0.45, *p* = 0.0034) (Figure 3B).

Importantly, total cerebral total hemoglobin (CHb), measured simultaneously using the same NIRS platform, remained stable before and after the passive post-exertional challenge in both hemispheres (Supplementary Figure S3). The absence of significant Hb changes indicates that the observed differences in cFTOE occurred without detectable alterations in CHb content or regional blood volume, supporting the interpretation that Hp phenotype is associated with differential cFTOE during physiological stress.

Although a similar numerical trend toward reduced cFTOE was observed in the left hemisphere (A1), these differences did not reach statistical significance (*p* > 0.05, Supplementary Figure S4). We present these data for completeness and transparency; however, statistically significant differences across Hp phenotypes were detected only in the right cerebral hemisphere in the present cohort. Likewise, no significant differences in cFTOE across Hp phenotypes were detected among SC participants in either hemisphere at baseline or following the passive post-exertional challenge (Supplementary Figure S5), indicating that these alterations are specific to the pathological context of LC rather than reflecting intrinsic physiological differences associated with Hp genotype. The observation that statistically significant differences were detected only in the right hemisphere is noteworthy given the concurrent differences in post-exertional cognitive responses across Hp phenotypes. Although the present study was not designed to establish causal relationships, the coexistence of reduced cFTOE and greater cognitive vulnerability in Hp2 carriers suggests that impaired oxygen utilization may represent a physiological signature accompanying post-exertional cognitive dysfunction.

Collectively, these findings demonstrate that individuals with LC carrying Hp2 phenotypes, particularly Hp2-2, exhibit reduced cFTOE following physiological stress despite stable cerebral hemoglobin measurements. The absence of comparable alterations in SC participants further suggests that these NIRS abnormalities represent disease-specific physiological responses associated with post-exertional cognitive vulnerability rather than constitutive differences in cerebral perfusion.

### 2.5. Structural and Quantitative Characterization of Haptoglobin Phenotypes

Having identified distinct cerebral physiological responses across Hp phenotypes, we next investigated whether these inherited Hp phenotypes differed in their structural organization or quantitative response to physiological stress. Chromatographic analyses confirmed the expected phenotype-specific organization of Hp oligomers. Tetrameric and pentameric retention time profiles were consistent with the inherited Hp1-1, Hp2-1 and Hp2-2 phenotypes and showed no evidence of major structural remodeling beyond their genetically determined organization (Supplementary Figures S6 and S7). Interestingly, tetramer retention times in both Hp2-1 and Hp2-2 individuals were significantly lower in previously characterized ME cohorts than in LC. This difference occurred despite the absence of a consistent relationship between oligomer percentage and retention time, suggesting that chronic disease progression may be associated with additional biochemical modifications affecting Hp molecular organization.

We next quantified circulating Hp concentrations before and after the passive post-exertional challenge. Plasma Hp levels increased significantly following the post-exertional challenge in LC participants but remained unchanged in SC controls (Supplementary Figure S8A,B), indicating that Hp mobilization represents a disease-specific response to physiological stress. As expected from the known genotype-dependent expression profile of Hp, individuals carrying the Hp2-2 phenotype exhibited lower circulating Hp concentrations than Hp1-1 or Hp2-1 participants at both baseline and post-exertional time points in both LC and SC cohorts (Supplementary Figure S8C–F).

To further investigate whether alterations in the heme-scavenging pathway contributed to the observed differences across Hp phenotypes, circulating low-density lipoprotein receptor-related protein 1 (LRP-1) concentrations were also evaluated (Supplementary Figure S9). Plasma LRP-1 levels were markedly lower in both LC and SC participants than in previously characterized pre-pandemic individuals with ME, whereas no significant difference was observed between LC and SC or among Hp phenotype groups within the LC cohort. These findings suggest that the pronounced soluble LRP-1 alterations previously identified in ME [5] are not yet established in LC and may instead emerge during disease progression.

Collectively, these findings demonstrate that while circulating Hp concentrations respond dynamically to physiological stress in LC, this response occurs without evidence of major oligomeric remodeling or differences in circulating LRP-1 across Hp phenotypes. These observations indicate that inherited Hp phenotype rather than structural reorganization underlies the observed biological stratification.

### 2.6. Hp Phenotypes Are Associated with Distinct Post-Exertional Metabolic Signatures

Having identified distinct cognitive, physiological, and Hp-associated biological profiles across Hp phenotypes, we next investigated whether these differences were accompanied by distinct post-exertional metabolic signatures. Plasma metabolomic profiles obtained from individuals with LC were compared across Hp1-1, Hp2-1, and Hp2-2 groups using a semi-untargeted liquid chromatography–mass spectrometry (LC-MS). A total of 111 metabolites were initially detected, of which 26 met predefined analytical quality criteria and were retained for subsequent analyses (Table 4). These metabolites were examined to identify phenotype-associated metabolic signatures; however, as these represent correlative observations, they should be interpreted as candidate biomarkers rather than as the causal mechanisms underlying resilience or vulnerability in LC.

#### 2.6.1. Metabolic Signatures Across Hp Phenotypes

**Figure 4:**
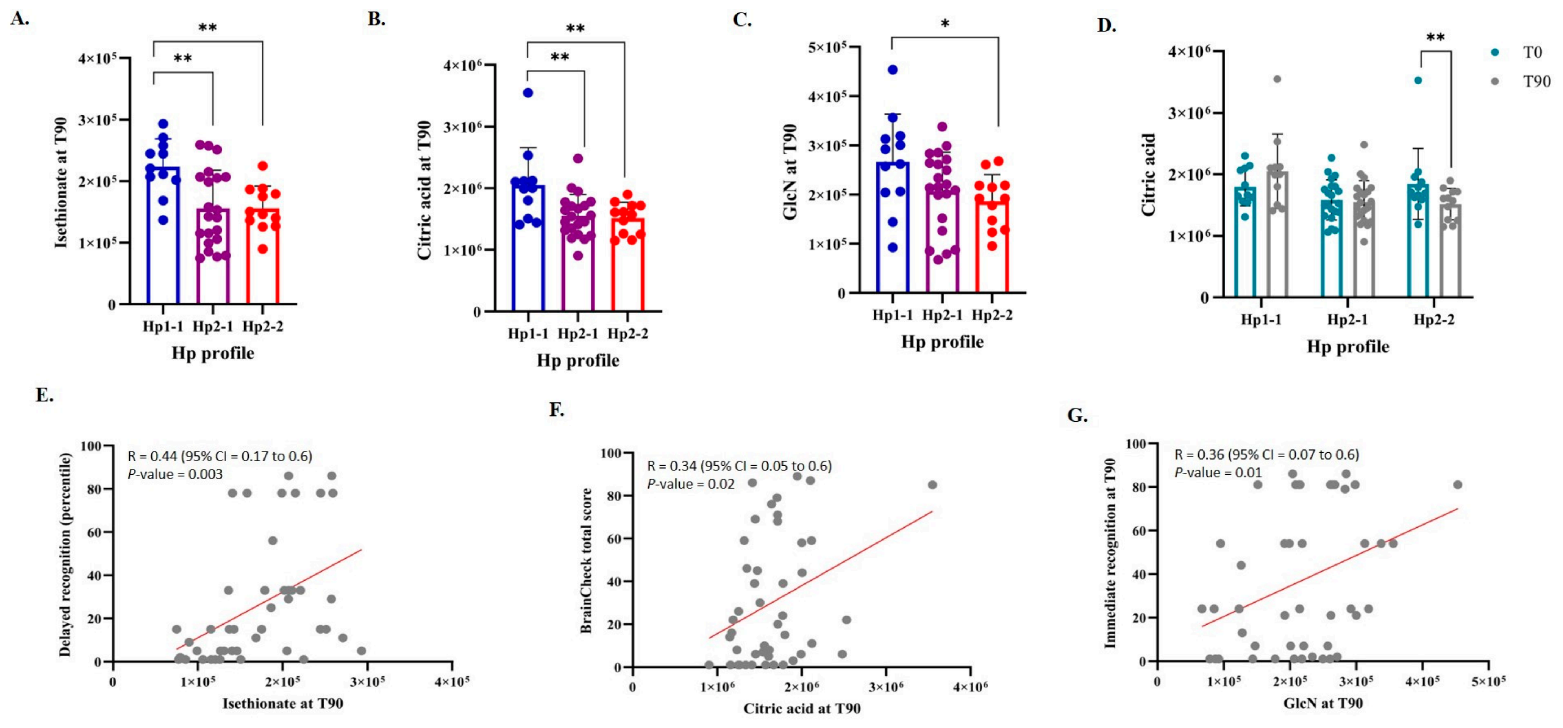
Metabolomic alterations across Hp phenotypes and their associations with cognitive performance following the post-exertional challenge. (**A**–**C**) Plasma levels of isethionate, citric acid, and GlcN at 90 min post-PEM (T90) across Hp phenotype groups, showing significantly lower levels of all three metabolites in Hp2 carriers compared with Hp1-1 individuals. Graphs for phenotypes Hp1-1, Hp2-1 and Hp2-2 are represented by the colors blue, purple and red respectively. (**D**) Comparison of citric acid levels at T0 vs. T90 demonstrating a consistent phenotype-associated trend, with an increase in Hp1-1 and a significant decrease in Hp2-2 participants. (**E**–**G**) Correlation between Isethionate, citric acid and GlcN levels and cognitive scores at T90. All data are represented as mean ± standard error of the mean. One-way ANOVA was performed to assess significant differences among the normally distributed groups, followed by the Tukey post hoc test for multiple comparisons. Kruskal–Wallis test followed by Dunn’s test for multiple comparisons was used for non-normally distributed groups. Paired *t*-tests were performed to assess within-group changes at T0 vs. T90. Pearson correlation was used to assess the correlation between the metabolites and the symptom severity. Results were considered significant at * *p*-value < 0.05 and ** *p*-value  <  0.01.

Analysis of T90 plasma samples identified significant differences in isethionate, citric acid, and glucosamine (GlcN) levels across Hp phenotypes (Figure 4A–C). Within the LC cohort, individuals carrying the Hp1-1 phenotype exhibited the highest post-exertional concentrations of these three metabolites, whereas progressively lower levels were observed in Hp2-1 and Hp2-2 patients. Effect sizes were large for isethionate (η^2^ = 0.25) and citric acid (η^2^ = 0.24) and moderate for GlcN (η^2^ = 0.15). After FDR correction, the associations remained significant for citric acid and isethionate but not for GlcN (Table 4). Given these results, citric acid and isethionate represent the most robust metabolic candidates for future investigation.

#### 2.6.2. Longitudinal Metabolomic Responses

In our exploratory analysis, baseline metabolomic analyses demonstrated largely comparable concentrations across Hp phenotype groups, with the exception of isethionate, which was significantly higher in Hp1-1 individuals than in Hp2-1 participants (Supplementary Figure S10). Longitudinal analyses further revealed distinct post-exertional metabolic responses across Hp phenotypes, with citric acid increasing significantly after PEM in Hp1-1 individuals while decreasing significantly in Hp2-2 participants. Similar trends were observed for isethionate and GlcN, although within-group changes did not reach statistical significance (Supplementary Figure S11).

#### 2.6.3. Associations Between Metabolomic Signatures and Cognitive Performance

To investigate potential relationships with cognitive function, correlations were assessed between metabolite concentrations measured at T90 and BrainCheck outcomes obtained immediately after the post-exertional challenge.

Higher citric acid concentrations were associated with higher total BrainCheck scores (Figure 4E), higher GlcN concentrations were associated with better immediate recognition performance (Figure 4F), and higher isethionate concentrations were positively associated with cognitive performance measures (Figure 4G). Other metabolites demonstrating significant differences after FDR correction, including fumaric acid, malic acid, and ribose, were not significantly associated with cognitive outcomes. Importantly, none of these metabolites exhibited significant post-exertional alterations in SC participants (Supplementary Figure S12), supporting the interpretation that the metabolic changes are associated with the LC post-exertional response and were not similarly observed in the SC comparator group. As these analyses are cross-sectional and exploratory, they suggest an association between metabolic state and cognitive performance that warrants future mechanistic validation.

#### 2.6.4. Summary of Metabolomic Findings

Taken together, these findings indicate that Hp phenotypes not only stratify symptom severity and cerebral physiology but are also associated with distinct metabolic responses to physiological stress, with Hp1-1 individuals exhibiting a more favorable post-exertional metabolic profile than Hp2 carriers.

## 3. Discussion

The present study demonstrates that inherited Hp phenotypes are associated with differential susceptibility to post-exertional cognitive dysfunction in LC and are accompanied by distinct cerebral oxygenation and metabolic signatures. While symptom severity, fatigue, and physical function differed across Hp phenotypes, the most clinically relevant observation was the marked vulnerability of Hp2-2 individuals to post-exertional cognitive decline. Together, these findings extend our previous observations in ME [5] and further support the concept that Hp phenotyping may serve as a candidate biological marker for identifying clinically meaningful LC endotypes with differential susceptibility to physiological stress.

Notably, Hp phenotype was not associated with a fixed domain-specific cognitive deficit detectable by cross-sectional comparisons at baseline or immediately following the post-exertional challenge. Rather, Hp phenotypes differentiated the dynamic cognitive response to physiological stress and cognitive trajectories over time. The Hp2-2 phenotype was associated with a significant acute post-exertional decline in global cognitive performance, whereas Hp2 allele carriers predominated among individuals with unfavorable longitudinal cognitive trajectories. Conversely, Hp1-1 was associated with relative cognitive resilience and longitudinal improvement. These findings suggest that Hp phenotype may act as a modifier of cognitive vulnerability to physiological stress and recovery rather than as a determinant of baseline or domain-specific cognitive impairment.

Importantly, the association between Hp phenotype and clinical severity remained significant after adjustment for age, sex, BMI and disease duration. Hp phenotype independently predicted physical function, fatigue severity, and PEM-related symptoms, whereas these conventional demographic and clinical variables did not significantly contribute to the regression models. This finding indicates that Hp phenotyping provides biologically relevant information beyond established clinical predictors and strengthens its potential utility as a candidate biomarker for biological stratification in future precision medicine applications.

Based on the present findings and our previous observations in ME, we further hypothesize that, at this earlier post-infectious stage, patients may still retain the capacity to mount an adaptive Hp response to physiological stress while maintaining the structural integrity of the Hp molecule [5]. In this conceptual framework, the expected gradient of Hp functional efficiency may become more apparent, with Hp2-2 individuals exhibiting the greatest vulnerability. We further propose that the divergent Hp-dependent patterns observed in ME and LC may reflect differences in post-infection timeframe, disease chronicity, and possibly the nature of the initiating viral trigger rather than fundamentally distinct disease processes. This conceptual model is hypothesis-generating and will require validation in prospective longitudinal and mechanistic studies. In ME, the greater severity observed in Hp2-1 individuals may be partly explained by failure of the hemopexin–LRP1 rescue axis, potentially driven by increased shedding of membrane-bound LRP1 and accumulation of soluble LRP1, thereby limiting effective heme/hemopexin clearance. By contrast, the present LC cohort showed a distinct post-exertional profile: circulating Hp increased after the passive challenge, CHb levels remained stable, Hp oligomeric organization was preserved, and circulating LRP-1 concentrations remained low relative to pre-pandemic ME patients with no significant differences observed across Hp phenotypes. Together, these observations suggest that the profound disruption of the hemopexin–LRP1 axis previously described in longstanding ME may not yet be established in LC, raising the possibility that compensatory scavenging Hb mechanisms may remain relatively preserved during earlier stages of post-infectious disease evolution. Although this model remains speculative, it provides a coherent framework in which LC and pre-pandemic ME can be viewed as related but temporally distinct states along a post-viral pathophysiological continuum. Within this framework, progressive exhaustion or dysregulation of compensatory scavenging pathways may contribute to the more hemolysis-prone phenotype previously observed in patients with longstanding ME.

The cognitive findings represent one of the principal contributions of the present study. Baseline cognitive performance was broadly comparable across Hp phenotypes; however, striking differences emerged following the application of a standardized physiological stress. Whereas SC participants exhibited the expected improvement associated with repeated testing, LC patients, particularly those carrying the Hp2-2 phenotype, displayed immediate post-exertional cognitive deterioration. Moreover, Hp1-1 individuals demonstrated more favorable longitudinal cognitive trajectories and were markedly underrepresented among participants exhibiting progressive cognitive decline. Together, these observations suggest that Hp phenotype primarily influences the capacity to maintain cognitive performance during physiological stress rather than baseline cognitive function itself. They also indicate that objective cognitive impairment may emerge during or immediately after exertion, preceding the delayed clinical manifestations that typically characterize PEM. Repeated cognitive assessments may be influenced by practice effects and test–retest variability. However, these factors are unlikely to fully explain the present findings. Whereas SC participants demonstrated the expected improvement in BrainCheck performance following repeated testing, consistent with a practice effect, participants with LC, particularly those carrying the Hp2-2 phenotype, exhibited post-exertional cognitive decline. The large effect size observed in the Hp2-2 subgroup, together with the consistency of the longitudinal cognitive trajectories, further supports the biological relevance of these differences across Hp phenotypes. Nevertheless, because cognitive performance was assessed immediately following the passive post-exertional challenge, our results likely capture early stress-induced cognitive alterations rather than the complete temporal evolution of PEM. We therefore hypothesize that these early cognitive alterations may represent a physiological precursor to the delayed clinical manifestations of PEM; however, this hypothesis requires confirmation through longitudinal studies incorporating serial post-exertional assessments. Future studies incorporating serial cognitive assessments throughout the post-exertional recovery period will be important to further characterize these temporal dynamics and distinguish biological responses from measurement variability.

The accompanying physiological findings provide further insight into the distinct cognitive responses observed across Hp phenotypes. LC patients carrying the Hp1-1 phenotype exhibited higher cFTOE following the post-exertional challenge, whereas Hp2 carriers, particularly Hp2-2 individuals, displayed reduced cerebral oxygen utilization despite stable cerebral Hb measurements. The preservation of cerebral Hb throughout the protocol indicates that these differences occur in the absence of detectable changes in cerebral blood volume and supports the interpretation that Hp phenotypes identify subgroups with distinct capacities for cerebral oxygen utilization during physiological stress. Statistically significant differences in cFTOE across Hp phenotypes were observed only in the right cerebral hemisphere, whereas the left hemisphere demonstrated a similar numerical trend that did not reach statistical significance. While the present study was not designed to establish causal relationships or hemispheric lateralization, the coexistence of reduced right cFTOE and greater cognitive vulnerability in Hp2 carriers suggests that altered oxygen utilization may represent a physiological signature accompanying post-exertional cognitive dysfunction. Based on the known biological properties of Hp phenotypes, we hypothesize that these differences may be associated with altered vascular and metabolic adaptation to physiological stress. Hp2-containing phenotypes, particularly Hp2-2, are characterized by reduced antioxidant capacity and less efficient hemoglobin/heme scavenging, which may increase susceptibility to oxidative stress and contribute to endothelial dysfunction and impaired microvascular oxygen delivery or utilization. However, oxidative stress, endothelial function, and cerebrovascular regulation were not directly assessed in the present study; therefore, these proposed mechanisms remain hypothesis-generating. Furthermore, although all NIRS measurements were acquired using a standardized bilateral prefrontal probe placement protocol, regional vascular variability and extracerebral tissue contributions may influence hemispheric oxygenation measurements. Accordingly, the apparent right-sided pattern should be interpreted cautiously and requires confirmation in larger independent cohorts using complementary neuroimaging approaches to determine whether it reflects a reproducible biological phenomenon.

The structural characterization of Hp further strengthens this interpretation. Tetrameric and pentameric organization remained remarkably stable following the post-exertional challenge, indicating that the acute increase in circulating Hp occurs independently of major oligomeric remodeling. Consequently, the biological stratification observed in LC is more likely to arise from genetically determined functional properties of Hp than from dynamic alterations in Hp architecture. Interestingly, the lower tetramer retention times observed in previously characterized ME cohorts compared with LC suggest that progressive post-translational or biochemical modifications may accumulate during chronic disease evolution without necessarily altering oligomeric composition. These observations raise the possibility that structural alterations emerge gradually over time and may contribute to the distinct biological landscape observed in longstanding ME.

The metabolomic findings provide potential insight into the physiological observations by demonstrating that Hp phenotypes are associated with coordinated rather than isolated adaptations to post-exertional stress. Hp1-1 individuals consistently exhibited higher post-exertional concentrations of citric acid, isethionate, and glucosamine together with greater cFTOE and better cognitive performance, whereas Hp2-2 individuals displayed the opposite profile. These convergent findings suggest that the inherited Hp phenotype is associated with differences in physiological adaptation to increased energetic demand. Citric acid, a central intermediate of the tricarboxylic acid cycle, is consistent with more efficient mitochondrial energy metabolism in Hp1-1 individuals [6,7,8], while reduced isethionate concentrations in Hp2 carriers are compatible with alterations in sulfur metabolism and redox homeostasis previously implicated in post-viral conditions [9,10,11]. Likewise, the lower glucosamine concentrations observed in Hp2-2 individuals may reflect differences in hexosamine biosynthetic pathway activity and protein O-GlcNAcylation, key regulators of cellular stress responses and mitochondrial function [12,13,14,15], although altered tissue uptake or metabolism cannot be excluded. Given the exploratory nature of these findings, we consider these metabolites candidate biomarkers rather than definitive mechanistic drivers. Citric acid and isethionate, which remained significant following FDR correction, represent the most robust candidates for further investigation, whereas the glucosamine finding should be considered exploratory. Collectively, these coordinated metabolic changes are compatible with a phenotype-dependent state of mitochondrial and endothelial stress. However, these interpretations represent biologically plausible hypotheses rather than experimentally established mechanisms and therefore require validation through targeted metabolomic, functional, and longitudinal mechanistic studies. The positive association between GlcN and recognition performance, together with its reported effects on Fibroblast Growth Factor 21 (FGF21) and Brain-Derived Neurotrophic Factor (BDNF) signaling, pathways implicated in metabolic adaptation and neuronal resilience [16,17,18], further identifies this pathway as a promising target for future mechanistic investigation. Importantly, all three metabolites were positively associated with post-exertional cognitive performance, and their coexistence with reduced cFTOE in Hp2-2 patients supports broader alterations in neuroenergetic adaptation previously implicated in LC [19,20]. However, because the present study is observational, these associations should be interpreted as complementary biological signatures rather than evidence of causal pathways. Current evidence does not support a direct regulatory effect of citric acid, isethionate, or glucosamine on circulating Hp or LRP-1. Instead, these metabolites are more likely to represent downstream manifestations of phenotype-dependent physiological adaptation, although glucosamine may indirectly intersect with this axis through hexosamine/O-GlcNAc-dependent regulation of inflammatory and cellular stress pathways. Collectively, these findings support a hierarchical model in which inherited Hp phenotype establishes the biological context within which adaptive responses to physiological stress unfold, manifesting as coordinated differences in cFTOE, metabolic flexibility, and ultimately susceptibility to post-exertional cognitive dysfunction. In this framework, the metabolomic alterations identified here should be viewed less as isolated biomarkers than as integrated biochemical signatures associated with differential physiological adaptation to post-exertional stress. A conceptual summary of this proposed hypothesis-generating framework is provided in Supplementary Figure S13.

From a translational perspective, the implications of these findings extend beyond biomarker discovery. Because Hp phenotypes are genetically determined and remain stable throughout life, they represent a robust biological characteristic that can be measured once and evaluated as a candidate marker for stratifying patients according to their susceptibility to post-exertional deterioration. Unlike dynamic circulating biomarkers that fluctuate with disease activity or treatment, Hp phenotyping provides a durable framework for biological classification that may facilitate interpretation of downstream physiological and molecular alterations and, pending prospective validation, contribute to risk stratification. Such an approach has important implications for precision medicine. Incorporating Hp phenotype into future clinical studies could reduce biological heterogeneity, improve cohort enrichment, and identify patients at increased risk of post-exertional cognitive decline who may benefit from closer monitoring or targeted interventions. Likewise, combining Hp phenotyping with stress-induced cognitive assessments, cerebral oxygenation measurements, and metabolomic profiling may facilitate the development of multimodal stratification algorithms capable of objectively characterizing LC endotypes and evaluating treatment responses. Beyond improving mechanistic understanding, this strategy could ultimately enhance patient selection for clinical trials and accelerate the development of personalized therapeutic approaches.

This study possesses several strengths, including its multidimensional integration of clinical, cognitive, physiological, structural, and metabolomic data using a standardized passive stress protocol that is well tolerated by patients with limited exercise capacity. Nevertheless, several limitations should be acknowledged. The relatively small sample size, particularly within the Hp1-1 and Hp2-2 phenotype subgroups, reduced statistical power for certain subgroup analyses. These findings should therefore be interpreted with caution as exploratory and hypothesis-generating. To improve the robustness of our analyses despite the modest subgroup sizes, we employed a rigorous statistical framework, including appropriate non-parametric tests for non-normally distributed data, effect size reporting, and FDR correction for metabolomic analyses. Nevertheless, these findings require validation in larger, independent cohorts. In addition, short-course COVID participants, who recovered rapidly from SARS-CoV-2 infection without persistent sequelae, provided the most relevant control group for comparison with LC participants experiencing persistent symptoms following the same infection. This comparison allowed us to examine biological differences associated with persistent illness versus successful recovery. Although never-infected healthy controls were not included in the present cohort, our previous study in ME included sedentary healthy controls from the same underlying population who had not been infected with SARS-CoV-2, providing a complementary reference for Hp phenotype distribution and post-exertional Hp-related responses. The semi-untargeted metabolomic approach captured only a subset of potentially relevant metabolic pathways, and the observational design precludes causal inference. Furthermore, although SC participants did not exhibit significant metabolomic changes during the post-exertional challenge, analyses stratified by Hp phenotype were not performed in this cohort, preventing determination of whether subtle genotype-dependent metabolic responses may also exist in healthy recovery. Finally, although BrainCheck assessments were administered in English to a predominantly French-speaking population, any language-related effects would be expected to influence all groups similarly and are therefore unlikely to account for observed differences in cognitive performance across Hp phenotypes. Future studies incorporating larger independent cohorts, longitudinal follow-up from acute infection through chronic disease, extended post-exertional sampling, and mechanistic metabolic analyses will be essential to validate and extend these findings.

## 4. Materials and Methods

### 4.1. Study Design and Participants

This prospective, longitudinal observational case–control study incorporated repeated-measures assessments and included 44 individuals with LC and 20 individuals referred to as SC, who recovered rapidly from SARS-CoV-2 infection within approximately two weeks, without persistent symptoms or sequelae and served as the control group. The SC cohort was intentionally selected to represent individuals who experienced confirmed SARS-CoV-2 infection but recovered without persistent symptoms, thereby providing the best comparator group to distinguish biological mechanisms associated with persistent post-infectious symptomatology from those related to viral infection itself. Participants were recruited through the clinical research program at the Azrieli Research Center at CHU Sainte-Justine (Montreal, QC, Canada). No participants reported SARS-CoV-2 reinfection during the study period.

Participants in the LC cohort were not hospitalized during their acute SARS-CoV-2 infection and reported persistent symptoms lasting at least three months after the initial infection, with a mean symptom duration of 10.3 ± 0.6 months at enrollment. The SC group consisted of individuals who recovered from SARS-CoV-2 infection without persistent symptoms following the acute phase. Participants with LC underwent three study visits at approximately three-month intervals. At each visit, standardized clinical assessments, cognitive testing, and biological sample collection were performed to evaluate longitudinal symptom trajectories and post-exertional responses.

To minimize potential confounding from overlapping post-infectious syndromes, individuals with pre-existing ME, fibromyalgia, multiple sclerosis, or a strong family history suggestive of these disorders were excluded. Additional exclusion criteria included major neurological, hematological, inflammatory, or vascular diseases; acute illness; unstable medical conditions; documented SARS-CoV-2 reinfection during follow-up; and the use of medications known to substantially affect cognitive function, cerebral oxygenation, or systemic metabolism.

The study was approved by the institutional research ethics board of CHU Sainte-Justine (protocol #MP-21-2021-3063), and all participants provided written informed consent before enrollment. The study was conducted in accordance with the Declaration of Helsinki and applicable regulations governing research involving human participants.

### 4.2. Clinical Symptom Assessment

Symptom severity and functional impairment were assessed at baseline before the post-exertional challenge using three validated self-reported instruments. General health status was evaluated using the SF-36, which measures physical and mental health-related quality of life across eight domains [21]. Fatigue severity was assessed using the MFI-20, encompassing physical fatigue, mental fatigue, reduced activity, reduced motivation, and general fatigue [22]. Core symptoms associated with PEM and related domains were evaluated using the DSQ [23]. To specifically assess exertional intolerance, analyses included DSQ items Q14 (“dead, heavy feeling after starting to exercise”) and Q18 (“physically drained or sick after mild activity”), which capture key features of PEM and were used as quantitative indicators of post-exertional symptom severity.

### 4.3. Post-Exertional Stress Induction Protocol

To evaluate physiological responses associated with PEM, participants underwent a standardized passive post-exertional challenge based on a previously validated protocol developed by Nepotchatykh et al. and subsequently applied in studies of ME and related post-viral conditions [24]. The challenge employed an ABR therapeutic massager device (Panacis Medical Ltd., Ottawa, ON, Canada), consisting of an inflatable cuff positioned around the upper arm that delivers cyclic mechanical compressions ranging from 0 to 4 psi at approximately 0.006 Hz.

Unlike conventional blood-pressure cuffs, which produce transient arterial occlusion, the ABR device generates sustained pulsatile mechanical stimulation over an extended period, inducing a standardized passive exercise. Participants underwent 90 min of continuous stimulation, after which biological samples and physiological measurements were repeated. Blood samples and physiological assessments were obtained at T0 and T90. This protocol was specifically designed to safely investigate post-exertional responses in individuals with limited exercise tolerance, including severely affected patients who may be unable to perform conventional exercise-based tests such as cardiopulmonary exercise testing (CPET).

### 4.4. Cognitive Assessment

Cognitive performance was evaluated using the BrainCheck digital cognitive assessment platform (BrainCheck Inc., Austin, TX, USA), a validated computerized tool for longitudinal monitoring of cognitive function [25]. Participants completed the assessment at T0 and T90 during the initial study visit. At each subsequent follow-up visit, assessments were performed at baseline only, resulting in three longitudinal evaluations per participant.

The BrainCheck battery comprises standardized neuropsychological tasks assessing attention, executive function, processing speed, and memory, including the Digit Symbol Substitution Test, Trail Making Tests A and B, Stroop Interference Test, and immediate and delayed recognition tasks. All assessments were administered in English. Automated scoring algorithms generated standardized domain-specific scores and an overall composite BrainCheck score while accounting for demographic variables, including age.

To minimize potential learning effects, participants completed a brief familiarization session before baseline testing using abbreviated versions of the tasks. Participants who completed all follow-up visits were subsequently categorized into distinct longitudinal cognitive trajectories based on the change in their BrainCheck composite score between the first (V1) and third (V3) visits. Cognitive trajectories were defined as deteriorated (decrease ≥11 points), stable (change between −10 and +10 points), or improved (increase ≥11 points). This threshold was derived from the distribution of longitudinal changes observed in the SC group, where fluctuations within ±10 points reflected expected measurement variability (Supplementary Figure S1).

### 4.5. Blood Collection and Plasma Preparation

Peripheral venous blood was collected at T0and T90, and during subsequent follow-up visits using EDTA-coated tubes. Samples were processed within a standardized timeframe (less than two hours) by centrifugation at 216× *g* for 10 min to separate plasma from cellular components. Plasma was aliquoted and stored at −80 °C until biochemical and metabolomic analyses to minimize freeze–thaw cycles. The same standardized collection and processing procedures were applied to all participants and time points to ensure sample consistency.

### 4.6. Cerebral and Peripheral Oxygenation Assessment Using NIRS

Given the evidence linking endothelial dysfunction and impaired oxygen utilization to neurocognitive symptoms in LC [26,27] and considering the differential antioxidant capacity and vascular protective effects associated with Hp phenotypes [28], cerebral and peripheral oxygenation were assessed to explore differences in oxygen utilization across Hp phenotypes during physiological stress. Cerebral oxygenation and hemodynamic parameters were evaluated using NIRS to estimate cerebral oxygen extraction during the post-exertional challenge.

Measurements were performed using the Masimo Root^®^ monitoring platform (Masimo Corp., Irvine, CA, USA) equipped with the O3^®^ Regional Oximetry and SET^®^ Pulse Oximetry modules. Regional cerebral oxygen saturation (rSO_2_) was continuously monitored using non-invasive NIRS sensors positioned bilaterally on the participant’s forehead according to the manufacturer’s recommendations. Simultaneously, peripheral arterial oxygen saturation (SpO_2_) was measured using integrated pulse oximetry, allowing estimation of cerebral oxygen extraction relative to systemic oxygen availability. cFTOE, an index of cerebral oxygen utilization, was calculated using the following equation [29]: cFTOE = (SpO_2_ − rSO_2_)/SpO_2_

Data acquisition followed a standardized temporal protocol. Baseline measurements were recorded during the 5 min immediately preceding the 90 min passive post-exertional challenge, and continuous monitoring was maintained throughout the stimulation period. A final 5 min recording was obtained immediately after completion of the challenge. To minimize transient fluctuations and motion artifacts, values were averaged over each 5 min recording interval before analysis. Measurements were collected bilaterally from both cerebral hemispheres, designated A1 (left hemisphere) and A2 (right hemisphere), to evaluate potential regional differences in oxygen extraction. Potential confounding factors, including skin pigmentation and forehead hair interference, were minimized through standardized sensor placement and signal quality controls provided by the Masimo platform. NIRS measurements were available for a subset of participants (LC, *n* = 22; SC, *n* = 19), as technical and logistical constraints during the early phase of recruitment amid the COVID-19 pandemic precluded data acquisition in all enrolled participants. Within this subset, the distribution of Hp phenotypes was monitored to ensure representative group comparisons for evaluating cerebral oxygen utilization across phenotype groups and between LC and SC participants.

In addition to rSO_2_, CHb was continuously monitored using the same Masimo Root^®^ platform equipped with the O3^®^ Regional Oximetry module as previously described [5]. Based on differential light absorption by oxygenated and deoxygenated hemoglobin, the device provides estimates of regional tissue oxygen saturation while simultaneously deriving an index of total CHb, reflecting the relative blood volume within the monitored cortical region. CHb signals were acquired continuously throughout the experimental protocol, including the baseline resting period, the 90 min passive post-exertional challenge, and the immediate recovery phase. For quantitative analyses, mean CHb values were calculated over predefined 5 min intervals corresponding to baseline (immediately preceding stimulation) and post-challenge (immediately following completion of the protocol). Bilateral recordings from the A1and A2 frontal regions were analyzed separately to evaluate potential hemispheric differences.

### 4.7. Haptoglobin (Hp) Phenotyping and Quantification of Circulating Hp and LRP-1

Hp phenotyping, quantification, and structural characterization were performed using high-performance liquid chromatography (HPLC) according to previously described protocols [5]. Plasma samples were analyzed to determine Hp phenotype distribution (Hp1-1, Hp2-1, and Hp2-2) based on differences in oligomeric structure and chromatographic retention time (RT). This HPLC-based approach provided an integrated assessment of Hp phenotype, circulating concentration, and oligomeric organization within a single analysis.

In addition to phenotypic classification, chromatographic profiles were used to quantify circulating Hp levels and characterize Hp proteoform composition. Relative Hp concentrations were estimated by integrating chromatographic peak areas corresponding to Hp oligomers. The HPLC approach also enabled analysis of higher-order Hp structures, including tetrameric and pentameric forms, based on their specific RTs and signal distribution. This integrated methodology allowed simultaneous evaluation of Hp phenotype, plasma abundance, and oligomeric structural features in each sample, facilitating comparisons between LC and SC participants as well as across Hp phenotype groups.

Circulating LRP-1 concentrations were measured in plasma using a commercial human LRP-1 ELISA kit (Abbexa Ltd., Cambridge, UK; Cat. No. abx152232) according to the manufacturer’s optimized protocols. This assay was performed to explore potential alterations in the hemopexin–LRP1 scavenging pathway and to compare LC findings with previously characterized pre-pandemic ME cohorts as previously described [5].

### 4.8. Plasma Metabolomic Profiling

Plasma polar metabolites were profiled by liquid chromatography–mass spectrometry (LC-MS) at Metabolomics Australia (University of Melbourne), an NCRIS-enabled facility. Samples were thawed on ice, and 20 µL of plasma was extracted by protein precipitation with 180 µL of ice-cold 1:1 (v/v) acetonitrile/methanol containing isotopically labelled internal standards (2 µM ^13^C_6_-sorbitol, 2 µM ^13^C,^15^N-AMP and 2 µM ^13^C,^15^N-UMP). Extracts were vortexed for 30 s, incubated for 10 min at 4 °C on a thermomixer, and centrifuged at maximum speed for 10 min at 4 °C. The supernatant was transferred to glass HPLC vials, and a pooled biological quality control (PBQC) sample was prepared by combining equal aliquots of all study-sample extracts.

Metabolites were separated by hydrophilic interaction liquid chromatography (HILIC) and detected on an Orbitrap high-resolution mass spectrometer (HILIC-LC-Orbitrap-MS) using a semi-untargeted profiling workflow. Study samples were randomized across the analytical run; internal standards were included in every sample, authentic metabolite standards were analyzed at the start of each batch, PBQCs were injected after every fifth study sample, and solvent blanks were run at the beginning and end of each batch. Raw data were processed and peaks integrated using El-MAVEN (v0.12.1), and metabolites were annotated against the Metabolomics Australia in-house polar-metabolite library (550 authentic standards) on the basis of accurate mass and retention time. Analytical performance was monitored using the isotope-labelled internal standards and PBQC samples; metabolites were retained if their coefficient of variation (CV) in the PBQCs was below 30%. Data diagnostics, log transformation (to correct for heteroscedasticity), and median normalization (to correct for inter-sample and sample-preparation variation) were performed in MetaboAnalyst 5.0 (https://www.metaboanalyst.ca/, accessed on 13 April 2026); principal component analysis confirmed tight clustering of PBQC samples, consistent with high analytical quality.

Semi-untargeted analysis was conducted on metabolites associated with key metabolic pathways, including oxidative stress regulation, mitochondrial energy metabolism, and glucose metabolism. For the redox category, analyses focused on metabolites associated with sulfur amino acid metabolism, glutathione-related pathways, and thiol balance due to their central role in redox homeostasis and antioxidant defense [30]. This was considered particularly relevant in the context of Hp phenotypes, which differ structurally through disulfide-linked polymerization and are closely associated with oxidative stress regulation, while thiol/disulfide balance itself is highly redox-sensitive and regulated by glutathione-dependent systems [31].

Metabolite concentrations were compared across Hp phenotype groups at T0 and T90 time-points to assess phenotype-associated metabolic responses to physiological stress. In the LC cohort, this included 11 participants with Hp1-1, 21 with Hp2-1, and 12 with Hp2-2 phenotypes.

### 4.9. Statistical Analysis

All statistical analyses were performed using GraphPad Prism software (version 8; GraphPad Software Inc., San Diego, CA, USA). Continuous variables are presented as mean ± standard error of the mean (SEM), unless otherwise specified. Data normality was assessed using the Shapiro–Wilk test together with visual inspection of Q–Q plots.

For between-group comparisons, normally distributed data were analyzed using Student’s *t*-test (two groups) or one-way analysis of variance (ANOVA) followed by Tukey’s multiple comparisons test, whereas non-normally distributed data were analyzed using the Mann–Whitney or Kruskal–Wallis test followed by Dunn’s post hoc test.

For repeated-measures analyses, normally distributed longitudinal data were analyzed using repeated-measures analysis of variance (RM-ANOVA), followed by Šídák’s multiple comparisons test, where appropriate. When normality assumptions were not satisfied, appropriate non-parametric tests were used.

Given the exploratory nature of this study and the restricted sample size, non-parametric tests were preferred whenever normality assumptions were not satisfied, as they provide a more robust approach for small samples and non-normally distributed data. Within-subject comparisons between T0 and T90 measurements were evaluated using paired *t*-tests or Wilcoxon signed-rank tests.

Associations between metabolic variables and clinical outcomes were assessed using Pearson or Spearman correlation analyses according to data distribution. To account for multiple testing in metabolomic analyses, false discovery rate (FDR) correction was applied using the Benjamini–Hochberg procedure.

Effect sizes were reported as eta squared (η^2^) for ANOVA and as Cohen’s *d* for pairwise comparisons. With interpretation based on conventional thresholds (η^2^: 0.01 small, 0.06 medium, 0.14 large; Cohen’s *d*: 0.2 small, 0.5 medium, 0.8 large). Statistical significance was defined as a two-sided *p*-value < 0.05.

To evaluate the independent association between Hp phenotype and clinical outcomes, multiple linear regression analyses were performed using SF-36 physical function, MFI-20 physical fatigue, and DSQ PEM scores as dependent variables. Models were adjusted for age, sex, body mass index (BMI), and disease duration. Hp phenotype was treated as an ordinal variable (Hp1-1 = 1, Hp2-1 = 2, Hp2-2 = 3), whereas sex was coded as a binary variable (female = 0, male = 1). Variance inflation factors (VIFs) were calculated to assess multicollinearity, and residual diagnostics were performed to verify model assumptions. Regression results are presented as unstandardized coefficients (β) with corresponding standard errors and 95% confidence intervals.

## 5. Conclusions

The present study identifies Hp phenotypes as promising candidates for biological stratification in LC, showing associations with differential susceptibility to post-exertional cognitive dysfunction. Rather than demonstrating that Hp phenotypes directly determine cerebral oxygenation or metabolic adaptation, our findings indicate that Hp phenotypes are associated with patient subgroups exhibiting distinct responses to physiological stress, accompanied by coordinated cerebral oxygenation and metabolomic signatures. The integration of clinical, cognitive, physiological, and metabolic observations supports an association between inherited Hp phenotype and differential responses to post-exertional stress, contributing to the marked heterogeneity observed in LC. While these findings provide a rationale for further evaluating Hp phenotyping as a tool for improving patient stratification and optimizing research design, its clinical utility, predictive performance, sensitivity, specificity, and external validity remain to be established through prospective validation in larger, independent cohorts. Moreover, the contrasting Hp dynamics observed between LC and other post-viral conditions raise the possibility that these phenotypes may reflect different trajectories of post-viral adaptation, a hypothesis warranting further prospective longitudinal investigation.

## Figures and Tables

**Table 1:** Clinical and demographic characteristics of study participants.

	LC (*n* = 44)	SC (*n* = 20)
Age (years)	42.6 ± 1.26	38.0 ± 2.42
Sex (male/female)	2/42	6/14
BMI (kg/m^2^)	26.7 ± 1.0	28.1 ± 1.6
Illness Duration (months)	10.3 ± 0.6	N/A
36-Item Short Form Health Survey (SF36)		
Physical score	33.3 ± 2.2 ****	87.7 ± 2.2
Mental score	45.3 ± 2.7 ****	78.9 ± 4.3
Multidimensional Fatigue Inventory-20 (MFI-20) Scores		
General Fatigue	18.2 ± 0.4 ****	8.7 ± 1.0
Physical Fatigue	17.5 ± 0.4 ****	8.0 ± 1.0
Reduced Activity	15.6 ± 0.5 ****	7.3 ± 0.8
Reduced Motivation	10.8 ± 0.5 **	7.7 ± 0.9
Mental Fatigue	15.5 ± 0.5 ****	7.9 ± 1.1
DePaul Symptom Questionnaire (DSQ) Scores		
Neuroendocrine, Autonomic and Immune Dysfunction score	33.4 ± 2.3 ****	9.8 ± 1.4
Cognitive Dysfunction score	53.2 ± 3.1 ****	11.8 ± 2.6
PEM score	64.0 ± 3.0 ****	12.2 ± 2.9
Sleep Disturbance score	50.1 ± 2.7 ****	20.1 ± 3.2

Values for the different SF-36, MFI-20 and DSQ categories are described as scores. All data are represented as mean  ±  standard error of the mean. A two-tailed Student’s *t*-test was used for the comparison between LC patients and SC. The questionnaire scores were significantly different between LC vs. SC. ** *p*-value  <  0.01; **** *p*-value  <  0.0001.

**Table 2:** Distribution of Hp phenotypes in LC and SC participants.

Hp Phenotype	LC (*n* = 44)	SC (*n* = 20)
Hp1-1	11 (25.0%)	4 (20.0%)
Hp2-1	21 (47.7%)	9 (45.0%)
Hp2-2	12 (27.3%)	7 (35.0%)

The table summarizes the distribution of Hp phenotypes (Hp1-1, Hp2-1, and Hp2-2) among LC and SC participants. Values are presented as *n* (%), where percentages are calculated within each study group.

**Table 3:** Distribution of Hp phenotypes according to cognitive trajectories.

Total BrainCheck Score During Follow-Up	Hp1-1	Hp2-1	Hp2-2
Deteriorated (*n* = 10)	10% (*n* = 1)	60% (*n* = 6)	30% (*n* = 3)
Improved (*n* = 13)	30.8% (*n* = 4)	53.8% (*n* = 7)	15.4% (*n* = 2)
Stable (*n* = 19)	31.6% (*n* = 6)	36.8% (*n* = 7)	31.6% (*n* = 6)

Cognitive alterations were defined based on longitudinal changes in the BrainCheck total composite score: Deteriorated (decrease relative to the baseline score), Improved (increased relative to the baseline score), and Stable (no change from baseline to the follow-up visit). Values are presented as percentages and absolute counts (*n*) within each clinical subgroup.

**Table 4:** Hp phenotype-dependent plasma metabolomic signatures after PEM.

Metabolites	*p*-Value	FDR
Glucose metabolism		
Ribose_HMDB0000283	0.004	0.04
Glucosamine_HMDB0001514	0.03	0.18
Ribitol_HMDB0000508	0.10	0.34
Stachyose_HMDB0003553	0.20	0.45
Rhamnose_HMDB0000849	0.20	0.42
Raffinose_HMDB0003213	0.24	0.45
3′-Sialyllactose_HMDB0000825	0.29	0.44
Glucose_HMDB0000122	0.37	0.51
Pyruvic acid_HMDB0000243	0.45	0.55
D-Lactic acid_HMDB0001311	0.58	0.64
Glycerol_HMDB0000131	0.92	0.92
TCA cycle		
Citric acid_HMDB0000094	0.003	0.02
Fumaric acid_HMDB0000134	0.009	0.03
Malic acid_HMDB0000156	0.02	0.03
Cis-Aconitic acid_HMDB0000072	0.07	0.10
Succinic acid_HMDB0000254	0.24	0.29
Oxoglutaric acid_HMDB0000208	0.40	0.40
Redox metabolism		
2-Hydroxyethanesulfonate_HMDB0003903	0.003	0.03
2-Hydroxybutyric acid_HMDB0000008	0.05	0.23
Hypotaurine_HMDB0000965	0.16	0.50
Cystine_HMDB0000192	0.18	0.41
Taurine_HMDB0000251	0.22	0.40
Methionine_HMDB0000696	0.36	0.54
Pyroglutamic acid_HMDB0000267	0.41	0.52
Methionine sulfoxide_HMDB0002005	0.46	0.52
Methylcysteine_HMDB0002108	0.71	0.71

Comparison of plasma metabolite concentrations across the three Haptoglobin phenotype groups (Hp1-1, Hp2-1, and Hp2-2) following the post-exertional stress challenge (T90). Metabolites were identified and quantified using LC-MS and categorized by functional pathway. Statistical significance was assessed using one-way ANOVA or Kruskal–Wallis tests as appropriate. Multiple testing was controlled using the Benjamini–Hochberg False Discovery Rate (FDR) procedure. Metabolites in bold (Citric acid, 2-Hydroxyethanesulfonate/isethionate, and Glucosamine) represent key candidates for phenotype-dependent exertional responses, with Citric acid and 2-Hydroxyethanesulfonate retaining statistical significance after FDR adjustment (FDR < 0.05). HMDB: Human Metabolome Database.

## Data Availability

The raw data supporting the conclusions of this article will be made available by the authors on request.

## Supplementary Materials

The following supporting information can be downloaded at https://www.mdpi.com/article/10.3390/ijms27157000/s1.

## Abbreviations

The following abbreviations are used in this manuscript:

ABR	Advanced Biomechanical Rehabilitation
BDNF	Brain-Derived Neurotrophic Factor
BMI	Body Mass Index
cFTOE	Cerebral Fractional Tissue Oxygen Extraction
CHb	Cerebral Total Hemoglobin
CHU Sainte-Justine	Centre Hospitalier Universitaire Sainte-Justine
CPET	Cardiopulmonary Exercise Testing
CV	Coefficient of Variation
DSQ	DePaul Symptom Questionnaire
EDTA	Ethylenediaminetetraacetic Acid
ELISA	Enzyme-Linked Immunosorbent Assay
FDR	False Discovery Rate
FGF21	Fibroblast Growth Factor 21
g	Gravity or Relative Centrifugal Force
GlcN	Glucosamine
Hb	Hemoglobin
HBP	Hexosamine Biosynthetic Pathway
HPLC	High-Performance Liquid Chromatography
Hp	Haptoglobin
Hz	Hertz
ICanCME	Interdisciplinary Canadian Collaborative Myalgic Encephalomyelitis (ICanCME) Research Network
LC	Long COVID
LC-MS	Liquid Chromatography-Mass Spectrometry
LRP-1	Low-Density Lipoprotein Receptor-Related Protein-1
ME	Myalgic Encephalomyelitis
MFI-20	Multidimensional Fatigue Inventory-20
MHz	Megahertz
NIRS	Near-Infrared Spectroscopy
O-GlcNAc	O-linked β-N-acetylglucosamine
PASC	Post-Acute Sequelae of SARS-CoV-2 Infection
PBQC	Pooled Biological Quality Control
PEM	Post-Exertional Malaise
Psi	Pounds Per Square Inch
R^2^	Coefficient of Determination
rSO_2_	Regional Cerebral Oxygen Saturation
RT	Retention Time
SARS-CoV-2	Severe Acute Respiratory Syndrome Coronavirus 2
SC	Short-Course COVID
SEM	Standard Error of the Mean
SF-36	Short Form-36 Health Survey
SpO_2_	Peripheral Oxygen Saturation
T0	Baseline Time Point (Pre-stimulation)
T90	Post-stimulation Time Point (90 min)
TCA	Tricarboxylic Acid (Cycle)
VIF	Variance Inflation Factor
V1	Visit 1
V3	Visit 3
β	Unstandardized Regression Coefficients
µL	Microlitre
η^2^	Eta Squared
