# Supplementary material for: Haptoglobin Phenotypes Stratify Post-Exertional Cognitive Dysfunction Associated with Altered Cerebral Oxygenation and Metabolic Signatures in Long COVID
Source: Int J Mol Sci. 2026 Aug 4;27(15):7000. doi: 10.3390/ijms27157000 (PMC13466397; doi:10.3390/ijms27157000)
Supplement: Supplementary file 1 [file ijms-27-07000-s001.zip › ijms-4425084-supplementary.pdf]

Haptoglobin Phenotypes Stratify Post-Exertional Cognitive Dysfunction Associated with Altered Cerebral Oxygenation and Metabolic Signatures in Long COVID

Atefeh Moezzi [1,2,3,4], Wesam Elremaly [2,3,4], Corinne Leveau [1,2,3,4], Anita Franco [2,3,4], Oleg Nepotchatykh [5], Christopher W. Armstrong [6] and Alain Moreau [1,2,3,4,7,*]

[1] Department of Biochemistry and Molecular Medicine, Faculty of Medicine, Université de Montréal, Montreal, QC H3C 3J7, Canada

[2] Viscogliosi Laboratory in Molecular Genetics of Musculoskeletal Diseases, Azrieli Research Center, CHU Sainte-Justine, Montreal, QC H3T 1C5, Canada

[3] Open Medicine Foundation ME/CFS Collaborative Center, CHU Sainte-Justine/ Universitéde Montréal, Mon-treal, QC H3T 1C5, Canada

[4] ICanCME Research Network, Azrieli Research Center, CHU Sainte-Justine, Montreal, QC H3T 1C5, Canada

[5] PO-Laboratories, Montreal, QC H4T 2A3, Canada

[6] Department of Biochemistry and Molecular Biology, Bio21 Molecular Science and Biochemistry Institute, Uni-versity of Melbourne, Parkville, VIC 3010, Australia

[7] Department of Stomatology, Faculty of Dentistry, Universitéde Montréal, Montreal, QC H3C 3J7, Canada

* Correspondence: alain.moreau.hsj@ssss.gouv.qc.ca

Supplementary File

Supplementary Table S1. Linear Regression of Physical Function Adjusted for Age, Sex, BMI and Disease Duration

Predictor Variable	SF-36 (Physical Functioning)	MFI-20 (Physical Fatigue)	DSQ (PEM Symptoms)
	β (SE)	β (SE)	β (SE)
(Constant)	85.76 (29.49) **	16.17 (7.32) *	-22.71 (68.73)
Hp Phenotype	-14.81 (4.87) **	3.37 (1.21) **	29.48 (11.35) **
Age	0.24 (0.42)	0.17 (0.10)	1.02 (0.98)
Sex (Male)	27.67 (16.82)	-4.95 (4.17)	-71.54 (39.21)
BMI	0.15 (0.72)	-0.08 (0.18)	0.03 (1.67)
Disease Duration	-0.90 (0.92)	0.18 (0.23)	1.12 (2.15)
Model R2	0.30	0.28	0.27
Model P-value	0.01	0.021	0.025

Results of three independent multiple linear regression models evaluating Hp phenotype as the primary predictor of physical health outcomes, including SF-36 (physical functioning), MFI-20 (fatigue), and DSQ (post-exertional malaise). All models were adjusted for age, sex, body mass index (BMI), and disease duration. Hp phenotype was modeled as an ordinal variable (1 = Hp 1–1, 2 = Hp 2–1, 3 = Hp 2–2). Sex was coded as 0 = female and 1 = male. Results are presented as unstandardized regression coefficients (β) with standard errors (SE). A negative β for SF-36 indicates reduced functional capacity with increasing Hp phenotype (toward Hp 2–2), whereas positive β values for MFI-20 and DSQ indicate greater fatigue and PEM severity. Model fit statistics (R^2) indicate that the models explain approximately 30% of the variance in outcomes. No significant associations were observed for age, sex, BMI, or disease duration, supporting the independence of the Hp phenotype effect. Statistical significance was defined as $^*p < 0.05$, $^{**}p < 0.01$.

Supplementary Table S2. Cognitive Performance in LC and SC at Baseline and Post-Exertion

	LC T0	LC T90	SC T0	SC T90
**BrainCheck total score**	39.8 ± 4.2^{a}	31.0 ± 4.5^{ab}	54.7 ± 5.8^{c}	63.2 ± 5.6^{bc}

Statistically significant differences were observed between LC at T0 and LC at T90, LC at T90 and SC at T90, and SC T0 and SC T90. Different superscript letters indicate statistically significant differences between groups (p < 0.05). Values are presented as mean ± SEM. Between-group comparisons were performed using independent t-tests, and within-group longitudinal comparisons were assessed using paired t-tests.

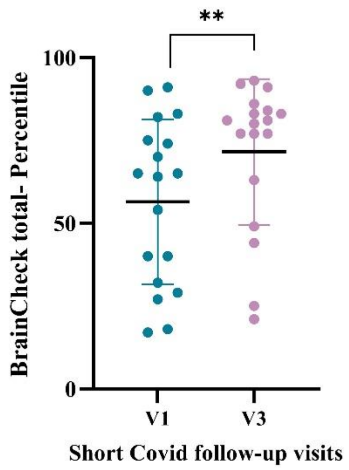

Supplementary Figure S1. Comparison of BrainCheck total scores between visit 1 and visit 3 in participants with short COVID (SC) (n = 18, minus two participants who missed visit 3 assessment). Individual paired data illustrate longitudinal changes in cognitive performance between visits. BrainCheck total scores increased significantly from V1 to V3, as assessed using a paired-samples t-test ($p < 0.01$), with a medium-to-large effect size (Cohen's d = 0.73).

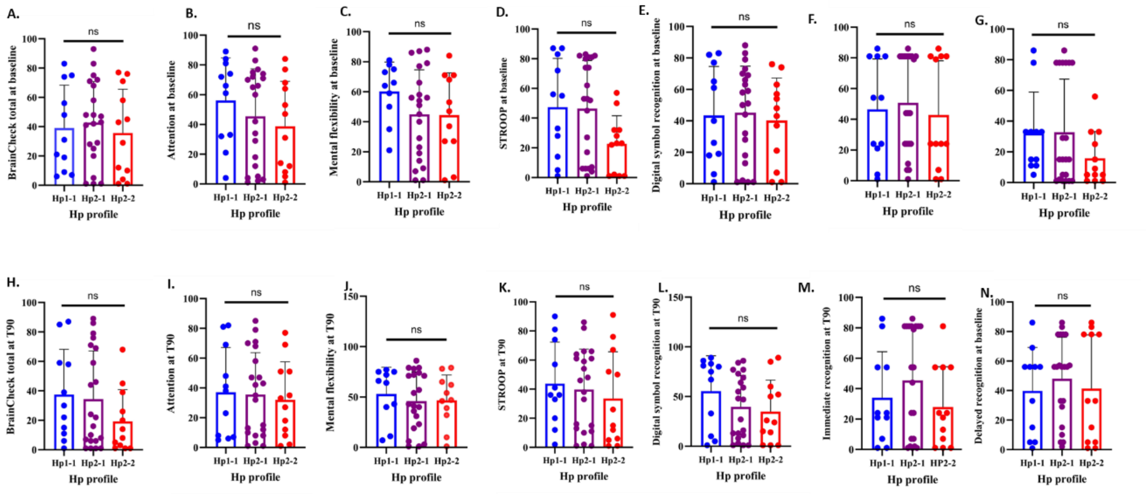

Supplementary Figure S2. Cognitive performance metrics across haptoglobin (Hp) phenotypes at baseline and following the post-exertional challenge. Cognitive performance was compared among Hp1-1 (blue), Hp2-1 (purple), and Hp2-2 (red) phenotypes. Panels A–G show baseline (T0) performance for (A) BrainCheck total, (B) attention, (C) mental flexibility, (D) Stroop, (E) digital symbol recognition, (F) immediate recognition, and (G) delayed recognition. Panels H–N show the corresponding

performance metrics at T90 following the post-exertional challenge. Data are presented as mean ± SEM. No statistically significant phenotype-dependent differences were observed for individual cognitive measures at either time point, although a consistent trend toward lower post-exertional performance was observed in the Hp2-2 group. Differences among Hp phenotypes were assessed using one-way ANOVA or the Kruskal–Wallis test, as appropriate. ns, not significant.

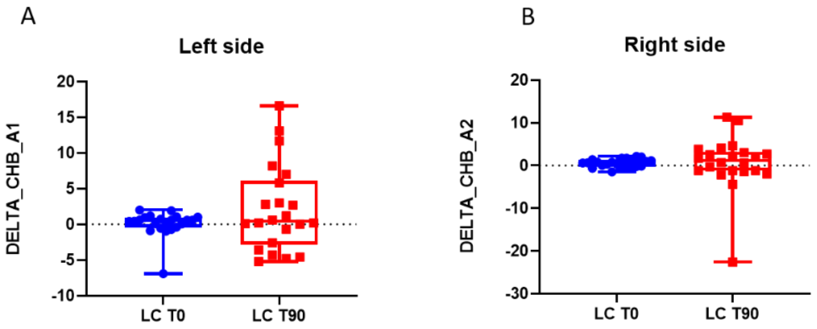

Supplementary Figure S3. The distribution of total cerebral hemoglobin (CHb) levels at baseline (T0) and post-exertional challenge (T90) in Long COVID (LC) patients. (A) Changes in total cerebral CHb for the left hemisphere. (B) Changes in total cerebral CHb for the right hemisphere. Data are presented as box-and-whisker plots indicating the median, interquartile range, and whiskers extending to the minimum and maximum values, with individual data points. Statistical comparisons between T0 and T90 were performed using paired t-test (ns, not significant).

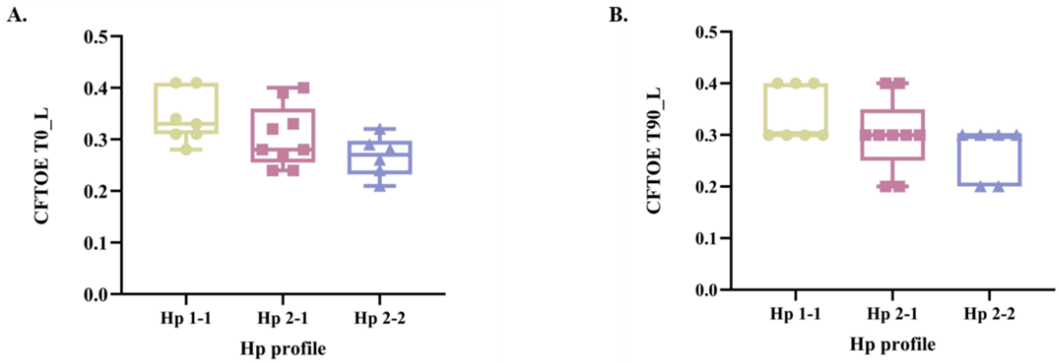

Supplementary Figure S4. Cerebral fractional tissue oxygen extraction (cFTOE) in left brain hemisphere (A1) across Hp phenotypes (Hp) at baseline (T0) and post-exertional challenge (T90) in Long COVID (LC) patients. (A) cFTOE in the left cerebral hemisphere (A1) across Hp phenotypes at T0. (B) cFTOE in the left cerebral hemisphere (A1) across Hp phenotypes at T90. Differences among the three Hp phenotype groups were assessed using one-way ANOVA. No statistically significant differences were observed among the groups.

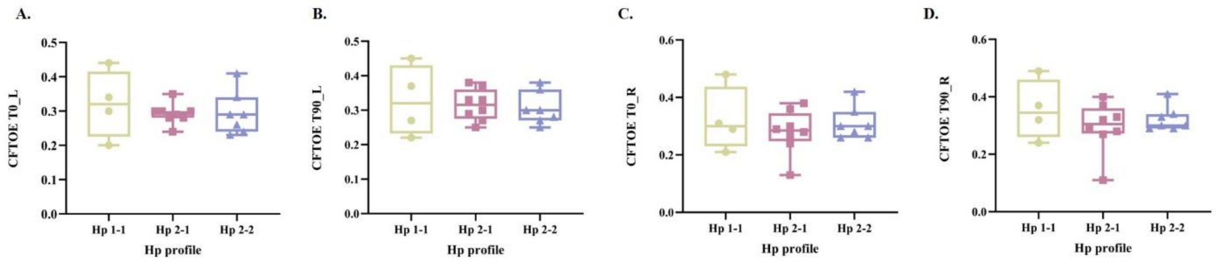

Supplementary Figure S5. Cerebral fractional tissue oxygen extraction (cFTOE) in Left (A1) and Right (A2) hemispheres across Hp phenotypes in short-course COVID (SC) group. (A) cFTOE in the left cerebral hemisphere at baseline (T0). (B) cFTOE in the left cerebral hemisphere following the post-exertional challenge (T90). (C) cFTOE in the right cerebral hemisphere at T0. (D) cFTOE in the right cerebral hemisphere at T90. No significant differences in cFTOE were observed among Hp phenotypes in either hemisphere at either time point. Differences among the three Hp phenotype groups were assessed using one-way ANOVA.

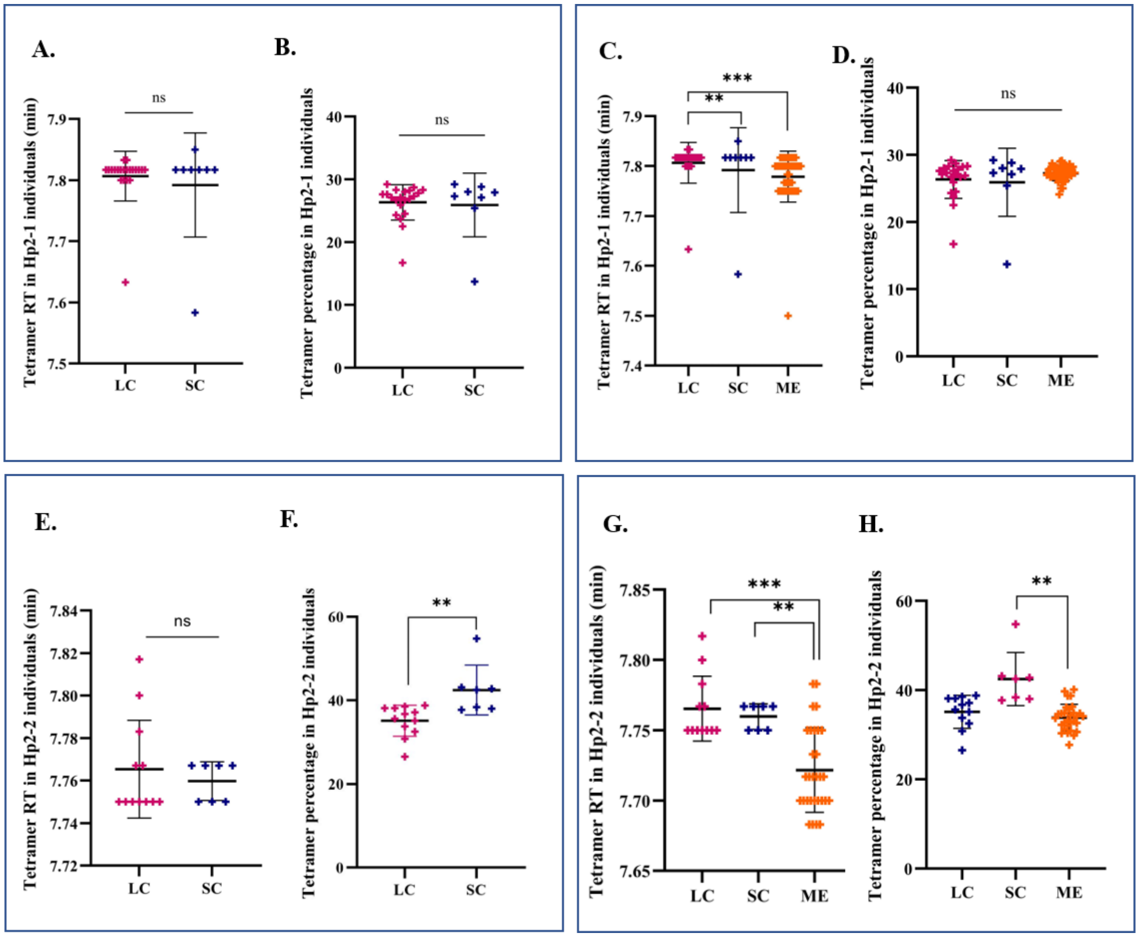

Supplementary Figure S6. Haptoglobin (Hp) tetramer distribution and retention time (RT) in Long COVID (LC), short-course COVID (SC), and myalgic encephalomyelitis (ME). (A–B) Tetramer RT and percentage in Hp2-1 individuals from the LC and SC groups. (C–D) Tetramer RT and percentage in Hp2-1 individuals across

LC, SC, and ME groups. (E–F) Tetramer RT and percentage in Hp2-2 individuals from the LC and SC groups. (G–H) Tetramer RT and percentage in Hp2-2 individuals across LC, SC, and ME groups. Tetramer RTs were significantly lower in ME than in LC for both Hp2-1 and Hp2-2, whereas no consistent relationship was observed between tetramer percentage and RT. Data are presented as mean ± SD. Two-group comparisons were performed using Student's t-test. Comparisons among three groups were performed using one-way ANOVA followed by Tukey's post hoc test for normally distributed data or the Kruskal–Wallis test followed by Dunn's multiple-comparisons test for non-normally distributed data. *p < 0.05, **p < 0.01, ***p < 0.001; ns, not significant.

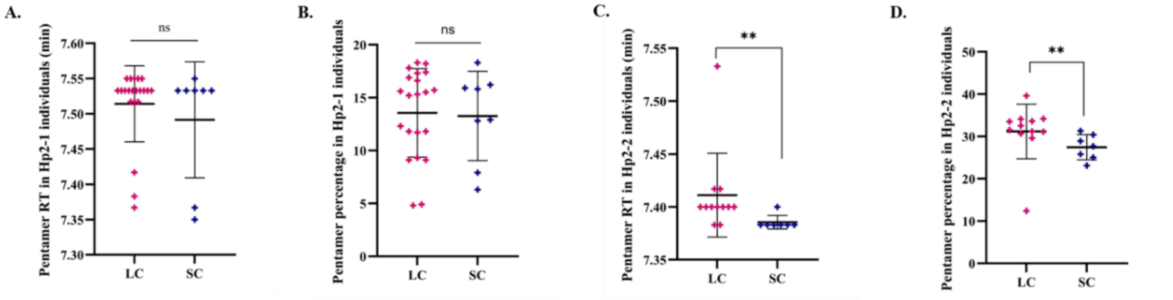

Supplementary Figure S7. Haptoglobin (Hp) pentamer distribution and retention time (RT) in Long COVID (LC) and short-course COVID (SC). (A-B) Pentamer RT and percentage in Hp2-1 individuals from LC patients and SC. (C-D) Pentamer RT and percentage in Hp2-2 individuals LC and compared to SC, showing significantly lower pentamer RT and pentamer percentage in SC compared with LC. Data are represented as mean ± SD. Student t-test was used for the two groups comparisons. Results were considered significant at **p-value < 0.01; ns, not significant.

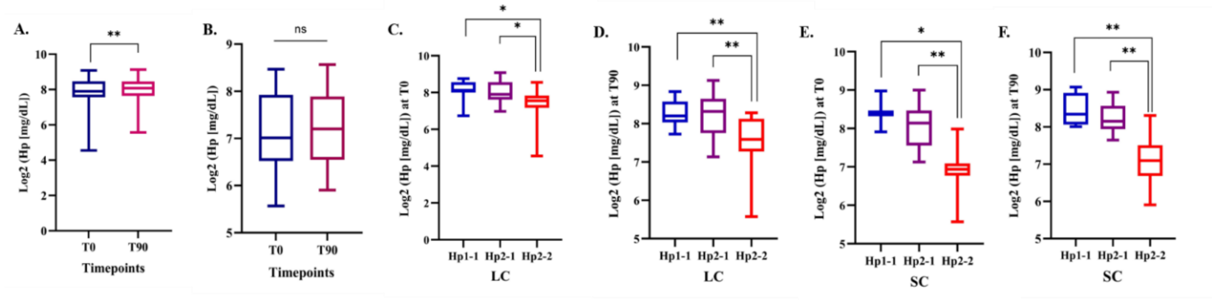

Supplementary Figure S8. Plasma haptoglobin (Hp) concentration before (T0) and following the post-exertional challenge (T90) in Long COVID (LC) and short-course COVID (SC) participants and across Hp phenotypes. (A) Plasma Hp concentrations in LC at T0 and T90. (B) Plasma Hp concentrations in SC at T0 and T90. Plasma Hp levels increased significantly following the post-exertional challenge in LC (Cohen's d = 0.43) but remained unchanged in SC. (C–D) Plasma Hp concentrations across Hp phenotypes in LC at T0 and T90, respectively. (E–F) Plasma Hp concentrations across Hp phenotypes in SC at T0 and T90, respectively. Consistent with the established genotype-dependent expression profile of Hp, individuals carrying the Hp2-2 phenotype exhibited lower plasma

Hp concentrations than Hp1-1 and Hp2-1 participants at both time points in both cohorts. Differences among Hp phenotype groups were assessed using one-way ANOVA (LC T0: $\eta^2 = 0.207$; LC T90: $\eta^2 = 0.264$; SC T0: $\eta^2 = 0.569$; SC T90: $\eta^2 = 0.557$), and within-group comparisons between T0 and T90 were assessed using paired t-tests. *$p < 0.05$; **$p < 0.01$; ns, not significant.

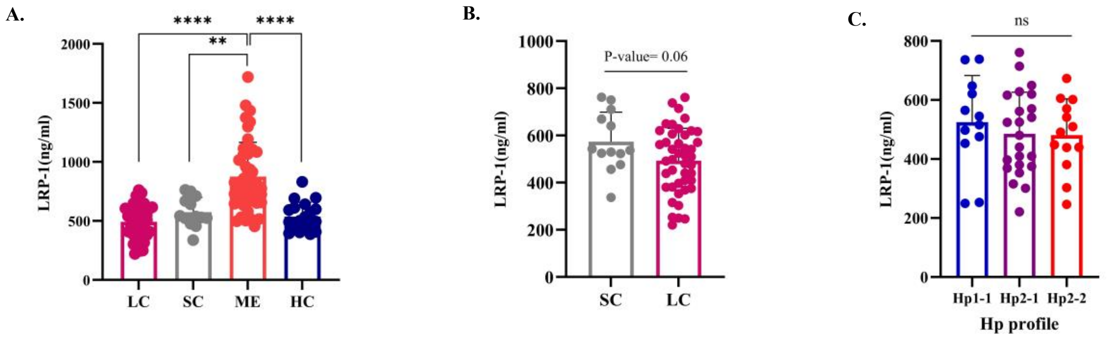

Supplementary Figure S9. Plasma LRP-1 concentrations across disease groups and haptoglobin (Hp) phenotypes. (A) Comparison of circulating LRP-1 concentrations among Long COVID (LC), short-course COVID (SC), myalgic encephalomyelitis (ME), and pre- pandemic healthy control (HC) groups. Significant differences were observed among groups, with a large effect size ($\eta^2 = 0.347$). (B) Comparison of circulating LRP-1 concentrations between LC and SC participants, showing no statistically significant difference. (C) Plasma LRP-1 concentrations across Hp1-1, Hp2-1, and Hp2-2 phenotypes within the LC cohort, showing no statistically significant phenotype-dependent differences. Data are presented as mean ± SD. Multiple-group comparisons were performed using one-way ANOVA followed by Tukey's post hoc test for normally distributed data or the Kruskal–Wallis test followed by Dunn's multiple-comparisons test for non-normally distributed data. Student's t-test was used for two-group comparisons. ***$p < 0.01$*; ****$p < 0.0001$; ns, not significant.

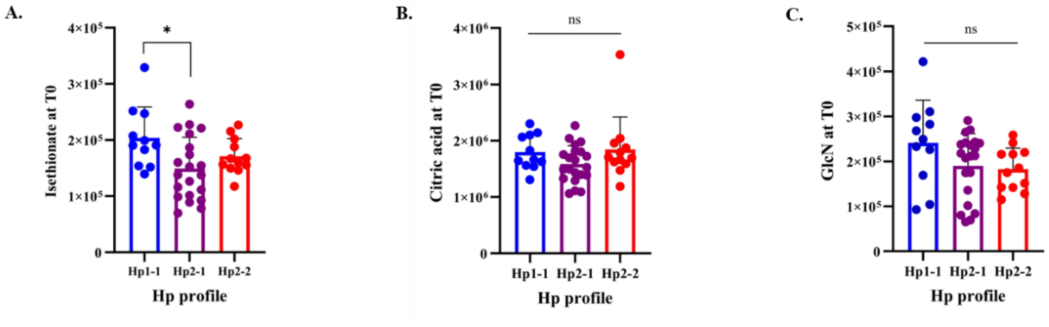

Supplementary Figure S10. Phenotype-dependent plasma metabolomic profiles at baseline (T0). (A–C) Plasma levels of (A) isethionate, (B) citric acid, and (C) Glucosamine (GlcN) across Hp1-1, Hp2-1, and Hp2-2 phenotypes. Isethionate levels were significantly higher in Hp1-1 than in Hp2-1 participants, whereas no statistically significant phenotype-dependent differences were observed for citric acid or GlcN. Data are represented as mean

± standard error of the mean. Differences among the three groups were assessed using one-way ANOVA followed by Tukey post hoc test for normally distributed data or Kruskal-Wallis test followed by Dunn's multiple comparisons test for non-normally distributed data. *p value < 0.05; ns, not significant.

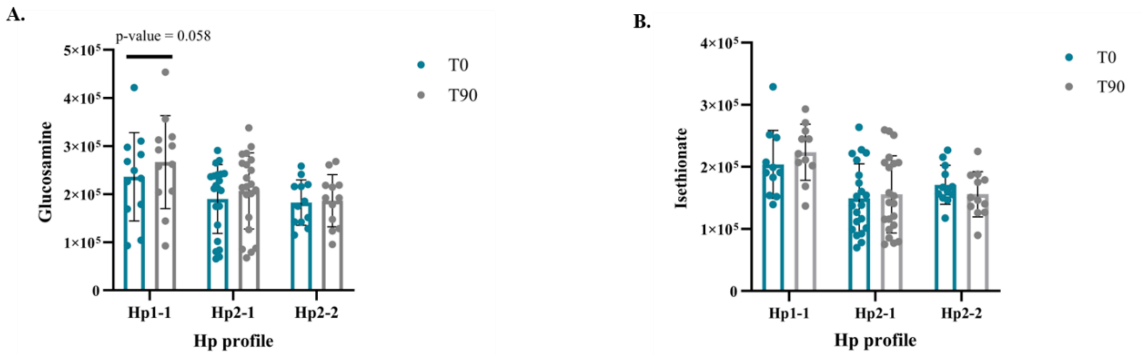

Supplementary Figure S11. Changes in plasma glucosamine and isethionate levels following the post-exertional challenge across haptoglobin (Hp) phenotypes in Long COVID (LC) participants. (A–B) Plasma levels of (A) glucosamine and (B) isethionate at baseline (T0) and following the post-exertional challenge (T90) across Hp1-1, Hp2-1, and Hp2-2 phenotypes. A phenotype-associated trend toward increased metabolite levels at T90 was observed in the Hp1-1 group, although the within-group changes did not reach statistical significance. Data are presented as mean ± SEM. Within-group differences between T0 and T90 were assessed using paired t-tests. ns, not significant.

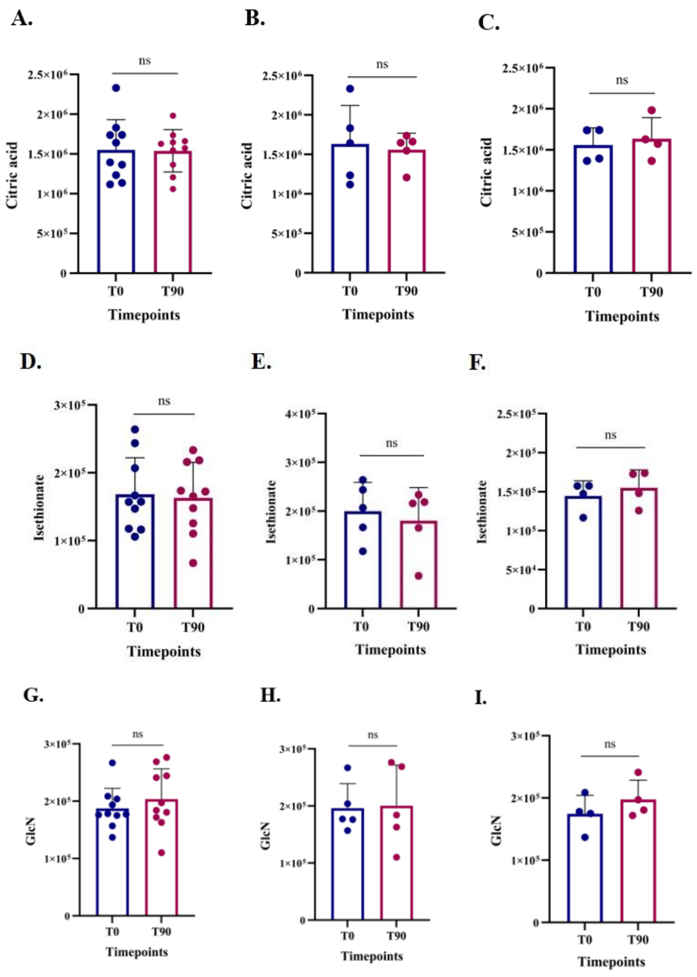

Supplementary Figure S12. Plasma metabolite levels before and after the post-exertional challenge in short-course COVID (SC) participants. Plasma levels of citric acid (A–C), isethionate (D–F), and glucosamine (GlcN) (G–I) were compared between baseline (T0) and post-exertional challenge (T90) in the overall SC cohort (n = 10) and in the Hp2-2 (n = 5) and Hp2-1 (n = 4) phenotype groups. No statistically significant changes were observed between T0 and T90 for any of the three metabolites in the overall SC cohort or within either Hp phenotype subgroup. Given the small subgroup sample sizes, paired comparisons were performed using the Wilcoxon signed-rank test. ns, not significant.

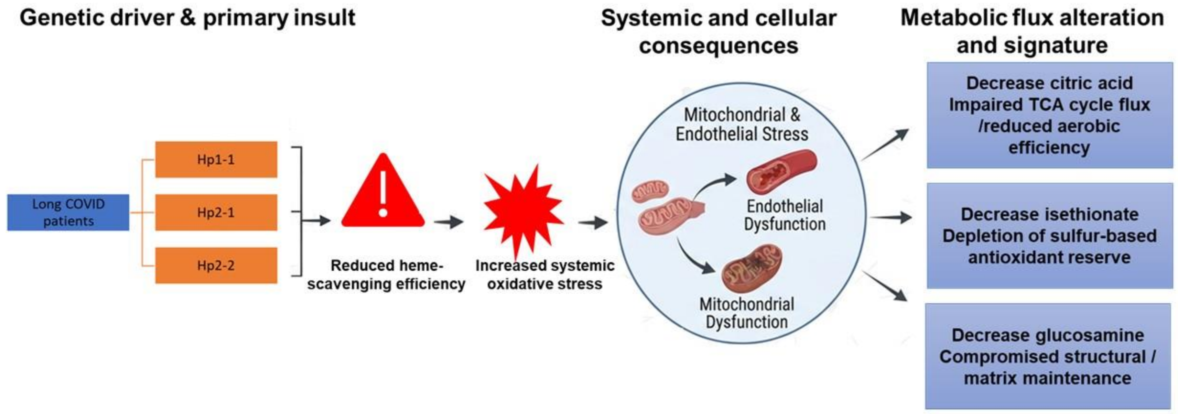

Supplementary Figure S13. Proposed hypothesis-generating framework linking haptoglobin (Hp) phenotypes to metabolic alterations in Long COVID (LC). This schematic illustrates a conceptual framework integrating Hp phenotype with the physiological and metabolic signatures observed in the present study. Hp2-containing phenotypes, particularly Hp2-2, are hypothesized to be associated with reduced heme-scavenging efficiency and greater susceptibility to oxidative stress, which may contribute to mitochondrial and endothelial dysfunction. These processes may be associated with the lower post-exertional levels of citric acid, isethionate, and glucosamine observed in Hp2 carriers. Reduced citric acid may reflect altered TCA cycle metabolism, while lower isethionate and glucosamine may be associated with alterations in redox homeostasis and cellular stress-response pathways, respectively. The proposed relationships are hypothesis-generating and do not establish causality; they provide a conceptual framework for future mechanistic and longitudinal investigation.
